# Immersion Frequency Optimisation and Species-Specific Metabolic Profiles of Colchicum autumnale and Colchicum bivonae in Temporary Immersion Systems

**DOI:** 10.3390/plants15111710

**Published:** 2026-05-31

**Authors:** Ivayla Dincheva, Ilian Badjakov, Vasil Georgiev, Radka Vrancheva, Ivan Ivanov, Liliya Georgieva, Atanas Pavlov

**Affiliations:** 1 Department of Agrobiotechnologies, Agrobioinstitute, Agricultural Academy, 8 Dr. Tsankov Blvd., 1164 Sofia, Bulgaria; ivadincheva@abi.bg (I.D.); ibadjakov@abi.bg (I.B.); liligeorgieva@abi.bg (L.G.); 2 Laboratory of Cell Biosystems, Institute of Microbiology, Bulgarian Academy of Sciences, 139 Ruski Blvd., 4000 Plovdiv, Bulgaria; vasgeorgiev@microbio.bas.bg; 3 Department of Analytical Chemistry and Physical Chemistry, University of Food Technologies, 26 Maritza Blvd., 4002 Plovdiv, Bulgaria; r_vrancheva@uft-plovdiv.bg; 4 Department of Organic Chemistry and Inorganic Chemistry, University of Food Technologies, 26 Maritza Blvd., 4002 Plovdiv, Bulgaria; i_ivanov@uft-plovdiv.bg

**Keywords:** *Colchicum* spp., GC-MS, metabolomics, tissue culture, plant biotechnology

## Abstract

Temporary immersion systems (TISs) are an advanced biotechnological platform for the large-scale cultivation of medicinal plants and the consistent production of high-value secondary metabolites. In this study, we evaluated three immersion regimes with stand-by periods of 4, 8, or 12 h, each paired with a 15-minute immersion period, to optimise shoot growth and colchicine accumulation in *Colchicum autumnale* L. and *Colchicum bivonae* Guss. The 4 h stand-by/15 min immersion regime yielded the highest growth index (*C. autumnale*: 0.75 ± 0.08; *C. bivonae*: 1.25 ± 0.03) and maximum colchicine content (*C. autumnale*: 0.19 ± 0.01 mg/g dry biomass; *C. bivonae*: 0.25 ± 0.02 mg/g dry biomass). Using gas chromatography-mass spectrometry (GC-MS), detailed metabolic profiling of cultures grown under this optimised regime was performed, resulting in the identification of 46 metabolites, including amino acids, organic acids, sugars, sugar alcohols, phenolic, and fatty acids. Volcano plot analysis revealed 11 upregulated and 5 downregulated metabolites in *C. autumnale* relative to *C. bivonae*. Significance analysis of metabolomics (SAM) identified 34 metabolites with statistically significant differences between two species. Hierarchical clustering and partial least squares discriminant analysis (PLS-DA) confirmed clear species separation, with Component 1 explaining 68.8% of the total metabolic variance. Glucose-6-phosphate (VIP = 2.01), citric acid (VIP = 1.85), asparagine (VIP = 1.67), and γ-aminobutyric acid (GABA; VIP = 1.52) were the primary biomarkers differentiating the species. These findings confirm that TISs provide an optimised environment for biomass accumulation and stable alkaloid biosynthesis in the Colchicum genus, with *C. bivonae* emerging as a promising candidate for biotechnological exploitation.

## 1. Introduction

The genus *Colchicum* (family Colchicaceae) comprises approximately 100–120 species of perennial geophytes distributed across Europe, North Africa, and Western Asia [1]. These plants have been recognised for centuries in traditional medicine, with their pharmacological significance primarily attributed to the production of phenethylisoquinoline alkaloids, most notably colchicine, demecolcine, and colchicoside [2,3]. These metabolites exert their therapeutic effects through high-affinity binding to tubulin, thereby inhibiting microtubule polymerisation and disrupting mitotic spindle formation [4]. This mechanism underpins their clinical applications in the treatment of acute gout flares, familial Mediterranean fever, Behçet’s disease, and various malignancies [5,6]. Colchicine remains one of the oldest anti-inflammatory drugs still in clinical use today, with expanding applications in cardiovascular medicine and oncology [7].

Recent advances have elucidated the colchicine biosynthetic pathway, revealing a complex cascade involving the coupling of two phenethylamine-derived units, followed by oxidative rearrangements and tropolone ring formation catalysed by cytochrome P450 enzymes and non-heme iron-dependent dioxygenases [8,9]. Despite these correlative findings, the pharmaceutical demand for colchicine has increased steadily, driven by expanded clinical indications [10]. However, conventional soil-based cultivation of Colchicum species faces considerable limitations, including slow growth cycles (one vegetative cycle per year), low seed germination rates, dependency on specific edaphic and climatic conditions, and significant inter-annual variability in alkaloid content [11,12]. Moreover, intensive harvesting of wild populations has led to conservation concerns, prompting the need for sustainable production alternatives.

To overcome these limitations, plant tissue culture has emerged as a sterile, reproducible alternative for the propagation and secondary metabolite production of medicinal plants [13,14,15]. Early studies on *Colchicum* tissue culture revealed that undifferentiated callus cultures often exhibit metabolic “silencing” or reduced biosynthetic capacity due to the loss of tissue-specific gene expression and cellular organisation [16,17]. Among the various in vitro techniques, temporary immersion systems (TISs) have emerged as a superior method for large-scale cultivation [18,19]. TIS bioreactors operate by periodically flooding plant material with liquid medium, followed by a stand-by period, allowing tissues to be exposed to the gaseous phase [20]. This cyclical regime confers several critical advantages: enhanced nutrient uptake, superior aeration and gas exchange, reduction in hyperhydricity (vitrification), and facilitated scalability [21,22].

Recent applications of TISs have demonstrated substantial improvements in biomass accumulation and secondary metabolite production across diverse medicinal plant genera, including *Catharanthus roseus*, *Panax ginseng*, and *Stevia rebaudiana* [23,24,25]. However, to date, no comprehensive study has evaluated the performance of TISs for *Colchicum* species or characterised the metabolic adjustments induced by this cultivation system.

Metabolomics, defined as the comprehensive analysis of low-molecular-weight metabolites in biological systems, provides a powerful platform for understanding the biochemical consequences of growth conditions and developmental stages [26]. Gas chromatography-mass spectrometry (GC-MS) offers advantages for primary metabolite profiling, enabling the simultaneous quantification of carbohydrates, amino acids, organic acids, and sugar alcohols in a single analytical run [27,28,29,30]. For alkaloid-producing plants, metabolomics has proven invaluable in mapping carbon and nitrogen flux into specialised metabolic pathways and identifying rate-limiting steps amenable to biotechnological intervention [31,32].

While previous studies have optimised TIS parameters for biomass accumulation in geophytes and others have measured colchicine content in field-grown Colchicum, no study to date has combined TIS optimisation with untargeted metabolomics to compare species-specific metabolic responses. The present study was undertaken to investigate changes in metabolite patterns in in vitro cultures of *C. autumnale* and *C. bivonae* cultivated in temporary immersion systems. These two species were selected due to their distinct geographical distributions and documented differences in alkaloid profiles [32,33]. To address the gap in the literature, the specific aims of the present study were: (i) to optimise TIS immersion frequencies (15 min immersion followed by 4 h, 8 h, and 12 h stand-by periods) for biomass growth and colchicine accumulation in two *Colchicum* species; (ii) perform comprehensive identification and quantification of primary and secondary metabolites using GC-MS-based metabolomics; (iii) compare the metabolic profiles of *C. autumnale* and *C. bivonae* using univariate and multivariate statistical approaches; and (iv) identify metabolites that differentiate the two species and to evaluate colchicine accumulation between them.

## 2. Results

### 2.1. Optimisation of Shoot Growth and Colchicine Accumulation in Temporary Immersion Systems

The number of introduced explants on semi-solid nutrient medium and the percentage of successfully sterilised material are presented in Supplementary Table S1. More than 82% of the explants were established under aseptic conditions. Bud enlargement was observed at the end of August, followed by the development of new flower stalks in September, in accordance with the natural biological cycle of the species [34]. Approximately 45 viable explants from each species were selected and transferred to the bioreactor system in November 2024.

**Figure 1:**
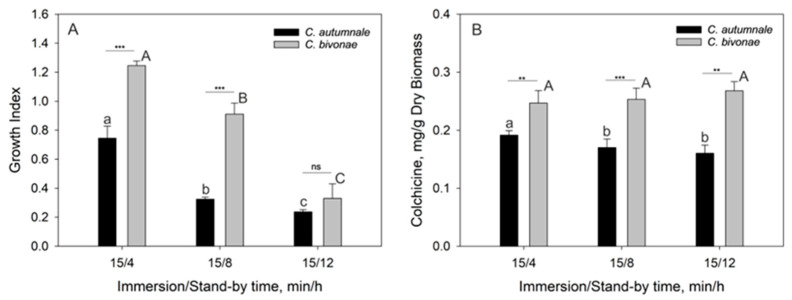
Growth index (**A**) and colchicine content (**B**) of *C. autumnale* and *C. bivonae* shoots cultivated in temporary immersion RITA^®^ systems with a 15 min submerging and 4 h, 8 h, and 12 h stand-by periods. Values represent the mean ± standard deviation (*n* = 3). Significant differences were evaluated by ANOVA followed by Tukey’s post-hoc test: lines indicate differences between species—ns, not significant; ** significant at *p* < 0.01; *** significant at *p* < 0.001; lowercase letters (a, b, c) indicate differences between regimes for *C. autumnale* at *p* < 0.05; uppercase letters (A, B, C) indicate differences between regimes for *C. bivonae* at *p* < 0.05.

To determine the optimal cultivation conditions for biomass proliferation and alkaloid production, *C. autumnale* and *C. bivonae* shoots were cultivated in temporary immersion systems (TISs) with immersion frequencies of 15 min immersion followed by 4 h, 8 h, and 12 h stand-by periods. The immersion frequency exerted a significant effect on both the growth index and colchicine accumulation in both species (Figure 1). It should be noted that an experiment was conducted under submerged conditions of cultivation (continuous immersion), but the shoots of both in vitro cultures turned brown and died shortly after the start of the experiment.

The regime with the shortest (4 h) stand-by period yielded the highest growth index, with values of 0.75 ± 0.08 for *C. autumnale* and 1.25 ± 0.03 for *C. bivonae* (Figure 1A). This regime also resulted in the highest colchicine accumulation, with *C. autumnale* reaching 0.19 ± 0.01 mg/g dry biomass (DB) and *C. bivonae* reaching 0.25 ± 0.02 mg/g DB (Figure 1B).

A two-level factorial analysis was performed to study the effects of the variables (immersion and stand-by periods) on the responses (growth index and colchicine production) and to optimise the process. The generated regression equations for *C. autumnale* (GI = 8.1 − 0.70 Stand-by − 0.48 Immersion + 0.043 Stand-by*Immersion; and Colchicine = − 0.610 − 0.1263 Stand-by + 0.0543 Immersion + 0.00816 Stand-by*Immersion) and *C. bivonae* (GI = 11.6 − 1.33 Stand-by − 0.66 Immersion + 0.081 Stand-by*Immersion; and Colchicine = −3.53 + 0.214 Stand-by + 0.251 Immersion − 0.0141 Stand-by*Immersion) exhibited high coefficients of determination (r^2^ = 85.09, r^2^ = 95.09, r^2^ = 95.56, and r^2^ = 76.22, respectively). The response optimiser function was employed to determine the optimal immersion and stand-by periods required to achieve the theoretical maximum growth index and colchicine production in both *Colchicum* species. In both cultures, the theoretical maximums were reached using an immersion frequency of 15 min immersion followed by 4 h stand-by periods (Supplementary Figure S1a,b). The differences between theoretical and experimental values for growth index and colchicine concentration were less than 15%, confirming the reliability of the optimisation model.

Both the growth index and colchicine content were significantly higher in *C. bivonae* under the 4 h stand-by regime relative to those in *C. autumnale* (*p* < 0.01 for the growth index; *p* < 0.001 for colchicine). To analyse the combined effect of immersion regime and the cultivated species on biomass accumulation (growth index) and colchicine production, a factorial analysis (species x immersion regime) was performed. The results are summarised in Table 1.

It was shown that the immersion regime had the most significant effect on the growth index, followed by the effect of species and the two-way interaction of (species* immersion). In contrast, for colchicine accumulation, the species had the most significant effect, whereas the two-way interaction was less significant, and the immersion frequency was not significant at all (Table 1, Supplementary Figure S2a–f). Based on these findings, all subsequent metabolomic analyses were performed on *C. autumnale* and *C. bivonae* shoots cultivated under the optimised TIS frequency of 15 min immersion and 4 h stand-by periods.

### 2.2. Comprehensive Metabolite Profiling Under Optimised TIS Conditions

GC-MS analysis was performed on biological replicates from all TIS regimes (4 h, 8 h, and 12 h stand-by) for both *C. autumnale* and *C. bivonae*. This analysis enabled the identification and quantification of 46 distinct metabolites, including essential amino acids, organic acids, sugars, sugar alcohols, phenolic compounds, fatty acids, and the target alkaloid colchicine. Species-specific differences in metabolite profiles were observed consistently across immersion frequencies. However, as the 4 h stand-by regime yielded the highest colchicine accumulation, it was the focus of the detailed metabolic interpretation presented below. The complete list of identified metabolites—including retention times, characteristic ions, and concentrations—is provided in Supplementary Table S1.

### 2.3. Differential Metabolite Expression Between Species

**Figure 2:**
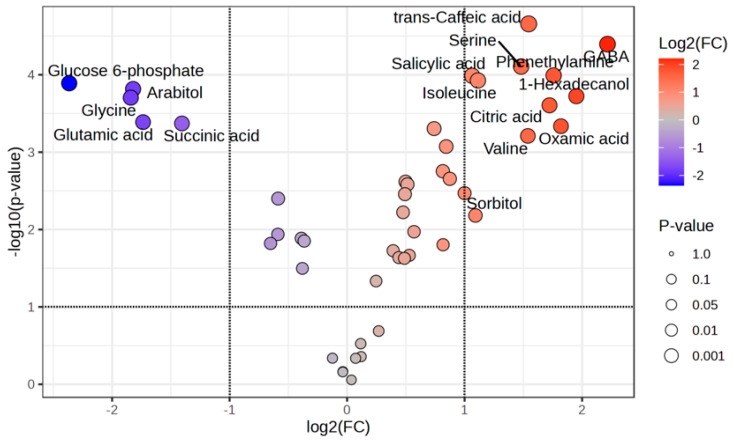
Volcano plot visualising the distribution of up- and downregulated metabolites in *C. autumnale* relative to *C. bivonae* shoots cultivated in temporary immersion systems with an immersion frequency of 15 min immersion and 4 h stand-by periods. The x-axis represents the log_2_ fold change (*C. autumnale*/*C. bivonae*), and the y-axis represents the −log_10_ *p*-value. Metabolites with a fold change > 2 and *p* < 0.05 are considered differentially accumulated. Upregulated metabolites in *C. autumnale* are shown in red; downregulated metabolites (i.e., enriched in *C. bivonae*) are shown in blue-purple; metabolites with no significant difference are shown in grey.

To visualise the distribution of differentially accumulated metabolites between the two species, a volcano plot was constructed by combining fold-change analysis with the Student’s *t*-test results (Figure 2). This analysis revealed that relative to *C. bivonae*, 11 metabolites were significantly upregulated in *C. autumnale*, while 5 metabolites were significantly downregulated.

### 2.4. Significance Analysis of Metabolomics (SAM)

**Figure 3:**
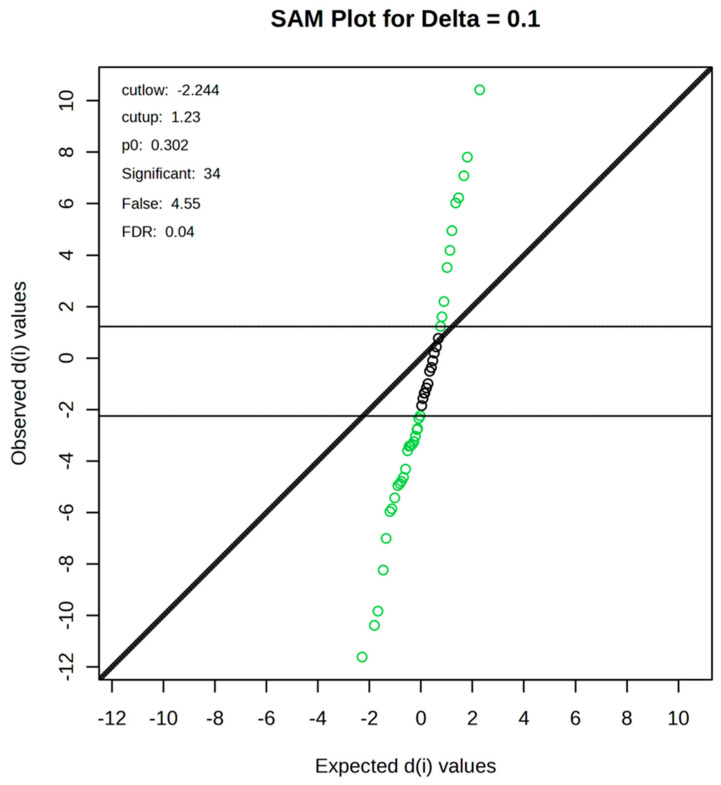
Significance analysis of metabolomics (SAM) of GC-MS-identified metabolites in *C. autumnale* and *C. bivonae* shoots cultivated in temporary immersion systems with an immersion frequency of 15 min immersion and 4 h stand-by periods.

To identify metabolites with statistically significant differences between the two species, significance analysis of metabolomics (SAM) was performed based on F-statistics (Figure 3). SAM identified 34 metabolites that differed significantly between *C. autumnale* and *C. bivonae* (false discovery rate < 0.05). This robust statistical approach confirmed that the majority of the identified metabolome (68%) exhibits species-specific regulation under TIS conditions.

### 2.5. Hierarchical Clustering of Species-Specific Metabolite Profiles

**Figure 4:**
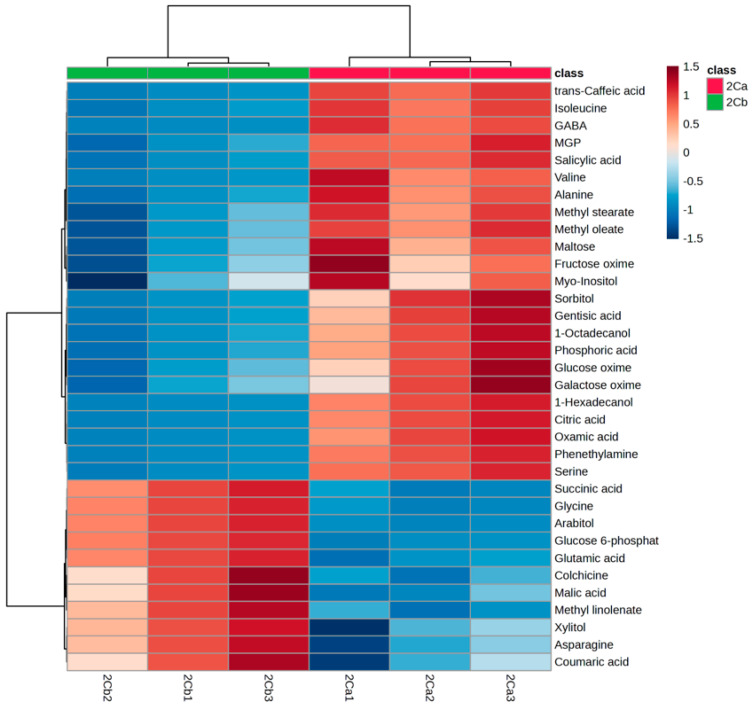
Hierarchical clustering heatmap of 34 metabolites identified as significant by SAM in *C. autumnale* and *C. bivonae* shoots cultivated in temporary immersion systems with a 15 min submerging and 4 h stand-by periods. Rows represent individual metabolites; columns represent biological replicates. Colour intensity reflects normalised abundance (row-scaled, with red indicating high relative abundance and blue indicating low relative abundance). The dendrogram at the top shows clear separation of the two species: *C. autumnale* (red) and *C. bivonae* (green). Ward’s clustering method with Euclidean distance was applied.

Hierarchical clustering analysis was applied to the 34 metabolites identified as significant by SAM to visualise the concentration differences between the two species (Figure 4). The heatmap revealed a clear separation of the two species into distinct clusters, with biological replicates clustering together, confirming the robustness of the metabolic differences. The heatmap further resolved species-specific metabolic signatures.

*C. bivonae cluster:* characterised by higher relative abundances of carbohydrates (sucrose, glucose, fructose, maltose), sugar alcohols (sorbitol, xylitol, arabitol), glucose-6-phosphate, asparagine, glycine, glutamic acid, and phenolic compounds (salicylic acid, gentisic acid, trans-caffeic acid). This cluster exhibits a metabolite profile consistent with enhanced carbon availability, elevated glycolytic intermediates, and nitrogen-rich compounds.

*C. autumnale cluster:* characterised by higher relative abundances of organic acids (citric acid, malic acid, succinic acid, glutaric acid, glyceric acid), stress-associated metabolites (GABA, pyroglutamic acid), and select amino acids (threonine, serine, valine). This cluster reflects a distinct metabolic profile centred on TCA cycle activity and stress readiness.

Notably, colchicine was more closely associated with the *C. bivonae* cluster, which is consistent with the quantitative finding of higher colchicine yields in this species under the optimised TIS regime.

### 2.6. Least Squares Discriminant Analysis

**Figure 5:**
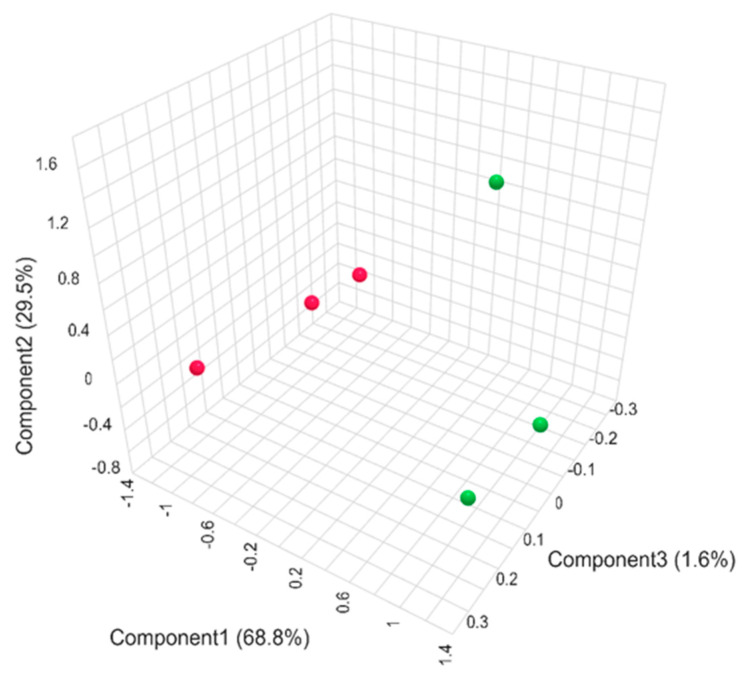
PLS-DA score plot of *C. autumnale* and *C. bivonae* metabolite profiles based on GC-MS data. Component 1 explains 68.8% of the total metabolic variance and clearly separates the two species. Component 2 explains an additional 29.5% of the variance. *C. bivonae* replicates (green) form a tight, homogeneous cluster; *C. autumnale* replicates (red) show greater dispersion. Each point represents an individual biological replicate.

To further synthesise the 46-metabolite dataset and validate the hierarchical clustering results, partial least squares discriminant analysis (PLS-DA) was employed. The PLS-DA score plot (Figure 5) revealed a distinct and robust separation between the two species along Component 1, which accounted for 68.8% of the total metabolic variance. *C. bivonae* samples clustered tightly on the positive side of Component 1, while *C. autumnale* samples occupied the negative side. Component 2 explained an additional 29.5% of the variance, further resolving intra-species variability.

Variable importance in projection (VIP) scores were used to identify the metabolites most influential in species discrimination. Glucose-6-phosphate achieved the highest VIP score (2.01), followed by citric acid (1.85), asparagine (1.67), and GABA (1.52). These metabolites, along with sucrose, malic acid, and pyroglutamic acid, were confirmed as the primary biomarkers differentiating the two species (Table 2).

The integration of PLS-DA and heatmap clustering revealed two fundamentally distinct metabolic profiles (Table 3).

These findings suggest that *C. bivonae* employs a proactive metabolic strategy, channelling carbon and nitrogen efficiently towards growth and secondary metabolite production. In contrast, *C. autumnale* appears to adopt a reactive or stress-primed strategy, maintaining high TCA cycle activity and accumulating stress-related metabolites, which may limit its capacity for alkaloid biosynthesis under identical TIS conditions.

## 3. Discussion

### 3.1. Optimisation of TIS Regimes for Colchicum Cultivation

The development of an effective micropropagation protocol is a complex task influenced by multiple interacting factors, including the type of plant material, the composition of the culture medium, and the cultivation conditions. In vitro propagation of *Colchicum* species remains challenging, and only limited information is available in the literature. The selection and preparation of suitable explants play a critical role in establishing successful in vitro cultures. Typically, tissues exhibiting active growth and a high physiological metabolic rate are preferred for culture initiation [35]. *Colchicum* species reproduce both by seeds and by corms. Vegetative propagation through corms is inherently slow, as each corm produces only one new corm (rarely more), while the original corm gradually senesces. Seed germination in *C. autumnale* is very low and highly irregular [36]. However, when plant in vitro cultures are used as technological matrices for the production of secondary metabolites, optimisation procedures are directed towards the accumulation of biomass (growth index, accumulated dry biomass), as well as the intensification of secondary metabolism, so as to achieve maximum yields of the target metabolite or group of metabolites.

The optimisation of immersion regimes in temporary immersion systems revealed that the immersion frequency of biomasses in the liquid medium significantly influences both biomass accumulation and secondary metabolite production in *Colchicum* species. The immersion frequency consisting of a 15 min immersion period and a 4 h stand-by period consistently produced the highest growth index, and colchicine yields for both *C. autumnale* and *C. bivonae*. This finding aligns with previous studies demonstrating that more frequent immersion cycles enhance nutrient availability and gas exchange, thereby promoting rapid growth and metabolic activity [18,19,37,38]. The reduced performance under longer stand-by periods (8 h and 12 h) likely reflects intervals of water and nutrient limitation that constrain both primary metabolism and alkaloid biosynthesis.

The superior performance of *C. bivonae* under the optimal regime indicated by a growth index of 1.25 relative to 0.75 for *C. autumnale* suggests species-specific variations in physiological responsiveness to TIS conditions. This disparity may reflect inherent differences in growth rates, tissue architecture, or nutrient uptake efficiency between the two species.

### 3.2. Integrated Metabolomic Composition and Species Differentiation

Unlike earlier TIS optimisation studies in geophytes that focused solely on growth parameters, the present work combines growth data with untargeted metabolomics, revealing species-specific metabolic signatures. Whereas previous Colchicum research relied on targeted approaches to measure only colchicine or a limited number of alkaloids, the GC-MS approach identified 46 distinct metabolites—including sugars, organic acids, amino acids, and phenolic compounds—providing a broader overview of carbon and nitrogen metabolism. Furthermore, it was demonstrated that *C. bivonae* and *C. autumnale* exhibited divergent metabolic profiles. Notably, these differences were consistently observed across all immersion frequencies. Additionally, correlations were identified between specific metabolites (e.g., glucose-6-phosphate) and colchicine yield, suggesting candidate biomarkers for future screening. Collectively, these advances establish a strong empirical basis for species-selective TIS scale-up and metabolome-informed process monitoring.

However, the underlying molecular mechanisms—such as gene expression, enzyme activities, or signalling pathways—that may link immersion frequency to metabolic changes were not investigated. Therefore, these findings should be interpreted as correlative and hypothesis-generating, providing a foundation for future mechanistic studies.

GC-MS-based metabolomics enabled the identification and quantification of 46 distinct metabolites, providing a detailed snapshot of the metabolic landscape of *C. autumnale* and *C. bivonae* under optimised TIS conditions. The combination of univariate (volcano plot, SAM) and multivariate (PCA, PLS-DA, hierarchical clustering) statistical approaches revealed robust species-specific metabolic signatures.

The PLS-DA score plot demonstrated clear separation between the two species, with Component 1 accounting for 68.8% of the total metabolic variance. This high percentage underscores that the species are distinguished not by minor fluctuations but by fundamental differences in core metabolic strategies. The tight clustering of *C. bivonae* replicates indicates a highly consistent and well-regulated metabolome, a critical attribute for industrial-scale bioprocessing where phenotypic stability is paramount [39]. Conversely, the broader dispersion of *C. autumnale* replicates suggests greater metabolic plasticity, potentially reflecting differential sensitivity to micro-environmental conditions within the TIS vessels.

### 3.3. Carbohydrate Metabolism

Carbohydrates represented the most significant fraction of the metabolome, accounting for the bulk of the osmotic and energetic pool. The coordinated accumulation of sucrose, glucose, fructose, and maltose in *C. bivonae* reflects active carbon assimilation and partitioning. Sucrose concentrations exceeding 112 mg/g DB in this species likely serve as a critical buffer for the energy-intensive synthesis of alkaloids, consistent with observations in other alkaloid-producing plants where carbohydrate availability correlates with secondary metabolite yields [27,29].

A pivotal finding was the significantly elevated concentration of glucose-6-phosphate (G6P) in *C. bivonae* (1.43 mg/g) relative to *C. autumnale* (0.28 mg/g), representing a fivefold higher concentration in the former species. As the entry point for both glycolysis and the pentose phosphate pathway, high G6P levels signify an intensified glycolytic flux that provides ATP and NADPH for reductive biosynthetic reactions [40,41]. The pentose phosphate pathway also generates erythrose-4-phosphate, a precursor for the shikimate pathway that feeds into phenylpropanoid and alkaloid biosynthesis [42].

### 3.4. Amino Acid Profiles and Nitrogen Assimilation

A fundamental divergence in nitrogen metabolism emerged as a defining feature separating the two species. *C. bivonae* displayed a “nitrogen-rich” profile characterised by high levels of asparagine (10.25 mg/g DB) and glycine (2.87 mg/g DB). Asparagine, with its high nitrogen-to-carbon ratio (2:4), serves as an ideal long-term storage and transport form of nitrogen. Its accumulation effectively “primes” the plant for the synthesis of nitrogen-containing secondary metabolites like colchicine, which contains three nitrogen atoms per molecule [43]. The correlation between asparagine levels and colchicine yields in *C. bivonae* suggests that nitrogen storage capacity may be a limiting factor for alkaloid biosynthesis, a concept supported by studies in other alkaloid-producing species [32]. In contrast, *C. autumnale* exhibited a signature dominated by pyroglutamic acid (25.4 mg/g DB) and GABA (4.73 mg/g DB). Pyroglutamic acid, a cyclised derivative of glutamic acid, is intimately linked to the glutathione cycle and accumulates under conditions of oxidative stress [44,45]. GABA, a four-carbon non-protein amino acid, integrates carbon and nitrogen metabolism and is a well-established stress-response metabolite that accumulates rapidly in response to various abiotic stresses [46,47]. The concurrent elevation of these compounds in *C. autumnale* suggests that this species maintains a metabolic configuration geared towards stress readiness, potentially at the cost of reduced carbon flux into specialised metabolism.

### 3.5. Organic Acid Flux and the TCA Cycle Dynamics

The profile of organic acids provided essential insights into respiratory efficiency and carbon skeleton availability. *C. autumnale* exhibited a “respiratory-heavy” profile, with citric acid concentrations (6.97 mg/g DB) exceeding twice the levels found in *C. bivonae* (2.72 mg/g DB). This suggests that *C. autumnale* shunts a greater proportion of its carbon pool into the tricarboxylic acid (TCA) cycle to sustain basal cellular maintenance and respiration. High TCA cycle activity has been associated with reduced allocation to secondary metabolism in various plant systems, as carbon skeletons are preferentially directed towards energy production rather than specialised metabolite biosynthesis [48,49].

The SAM analysis identified citric acid as one of the most significant discriminators between the species (Figure 3), and the volcano plot confirmed its strong upregulation in *C. autumnale* (Figure 2). This consistent finding across multiple statistical approaches reinforces the central role of TCA cycle flux in defining the metabolic identity of *C. autumnale*.

### 3.6. Secondary Metabolism in TISs

The target alkaloid, colchicine, was identified in all samples, demonstrating that the optimised TIS regime (15 min immersion and 4 h stand-by periods) provides a stable environment for specialised metabolism. This finding is particularly significant given that undifferentiated in vitro cultures often exhibit metabolic silencing due to the loss of tissue-specific gene expression [16,50]. The optimised gaseous exchange and nutrient availability of TISs likely prevent this phenomenon, consistent with recent reports on the efficacy of TISs for maintaining biosynthetic capacity in medicinal plants [23,24].

A consistent disparity in alkaloid accumulation was observed: *C. bivonae* exhibited superior biosynthetic capacity, reaching concentrations of 0.25 ± 0.02 mg/g DB relative to 0.19 ± 0.01 mg/g DB in *C. autumnale*. This 32% higher yield suggests that *C. bivonae* possesses a more efficient enzymatic “pull” through the phenethylisoquinoline pathway under TIS conditions. The coordinated accumulation of phenolic compounds (salicylic acid, gentisic acid, trans-caffeic acid) in *C. bivonae*, as visualised in the heatmap (Figure 4), further indicates activation of the broader phenylpropanoid pathway, which shares upstream precursors with alkaloid biosynthesis [51,52].

### 3.7. Biomarker Identification and Biotechnological Implications

The integration of volcano plot analysis, SAM, and VIP scores identified a set of robust biomarkers that differentiate the two species. Glucose-6-phosphate (VIP = 2.01), citric acid (VIP = 1.85), asparagine (VIP = 1.67), and GABA (VIP = 1.52) emerged as the most discriminatory metabolites. These biomarkers lie at the critical intersection of primary carbon metabolism (G6P, citric acid) and nitrogen assimilation (asparagine, GABA), underscoring that species-specific differences in these core pathways ultimately dictate metabolic flux towards secondary metabolites.

From a biotechnological perspective, *C. bivonae* emerges as a promising candidate for large-scale colchicine production due to:

Higher alkaloid yields: 0.25 mg/g DB colchicine under the optimised regime.

Metabolic consistency: tight clustering in multivariate analyses indicates phenotypic stability.

Efficient resource allocation: high G6P and asparagine levels support sustained secondary metabolism. The identification of G6P as the top-ranked biomarker (VIP = 2.01) suggests that monitoring this metabolite could serve as a rapid indicator of biosynthetic capacity in *Colchicum* cultures, thereby enabling real-time process optimisation in bioreactor systems [53,54].

### 3.8. Limitations and Future Directions

Several limitations of this study should be acknowledged. First, while 46 metabolites were successfully identified and distinct species-specific differences were observed, the underlying molecular mechanisms, such as gene expression, enzyme activities, or signalling pathways that may link immersion frequency to these metabolic changes were not investigated. Second, the metabolomic data consisted of steady-state measurements, which do not measure metabolic flux or establish direct causality. Consequently, observed associations, such as the relationship between elevated G6P levels and higher colchicine yield, should be interpreted as correlative and hypothesis-generating rather than mechanistic. Finally, although metabolic differences were consistent across all treatments, this study evaluated only three immersion regimes (4 h, 8 h, and 12 h stand-by periods), providing a relatively narrow window into the potential of the system.

To build on these findings and move towards full biotechnological control of Colchicum alkaloid production, a coordinated, multi-pronged research strategy is required. Future studies should prioritise systematic parameter fine-tuning by varying immersion frequency and duration across a finer gradient (e.g., intervals of 2 h, 6 h, and 10 h) to define optimal conditions for growth and biosynthesis more precisely. This should be paired with time-series metabolomics, sampling every 7–14 days to map metabolic flux during culture development and identify critical temporal windows for alkaloid accumulation. Beyond expanded profiling, multi-omics integration will be essential to bridge the gap between observation and mechanism. By combining metabolomics with transcriptomics, researchers can identify differentially expressed genes within the colchicine biosynthetic pathway, such as TYDC, OMT, and CYP71FB1 and pinpoint regulatory nodes controlling carbon and nitrogen partitioning. Furthermore, employing enzyme activity assays and targeted signalling inhibitors will help determine the specific pathways that mediate the effects of immersion frequency on metabolism. Finally, as laboratory-scale processes are refined, research must address industrial scalability, transitioning from RITA^®^ bioreactors to larger-scale TISs while exploring the use of biotic and abiotic elicitors to further maximise production within the optimised framework.

Notwithstanding these limitations, this study provides a detailed metabolomic characterisation under optimised TIS conditions and serves as a strong basis for further integrated research. The findings point towards logical next steps for the biotechnological exploitation of Colchicum alkaloid production.

## 4. Materials and Methods

### 4.1. Plant Material and Establishment of In Vitro Cultures

In compliance with Permit No. 965/27.01.2023 issued by the Ministry of Environment and Water of Bulgaria, corms of *C. autumnale* and *C. bivonae* were collected from two natural habitats (Supplementary Table S1) considered characteristic and representative for each species. All accessions were taxonomically authenticated by Dr. Ivanka Semerdjieva (Agricultural University, Plovdiv, Bulgaria/Institute of Biodiversity and Ecosystem Research, Bulgarian Academy of Sciences). Voucher specimens were deposited in the herbarium of the Agricultural University, Plovdiv (SOA) under the following accession numbers: *C. bivonae*—063585 and *C. autumnale*—063586.

Corms of *C. autumnale* and *C. bivonae* were cleaned by removing outer scales, washed with 70% (*v*/*v*) ethanol for 1 min, and air-dried in a laminar flow hood (Figure S3). Surface sterilisation was performed by immersing the corms in a 50% (*v*/*v*) commercial bleach solution (containing ~2.5% active sodium hypochlorite) supplemented with two drops of Tween^®^ 20 per 100 mL for 20 min, followed by three rinses with sterile distilled water. From the disinfected corms, explants containing meristematic tissue (main and lateral buds) were excised (Figure S4) and subjected to a second sterilisation step in 50% (*v*/*v*) bleach for 20 min, followed by three rinses with sterile distilled water. After drying on sterile filter paper, the explants were cultured in test tubes containing full-strength Murashige and Skoog (MS) medium [55] supplemented with 0.5 mg/L 6-benzylaminopurine (BAP, Duchefa Biochemie B.V., Haarlem, The Netherlands) and 30 g/L sucrose. The pH of the medium was adjusted to 5.7 ± 0.1 before autoclaving at 121 °C for 20 min. Cultures were incubated at 25 ± 2 °C under a 16 h photoperiod (cool-white, fluorescent lights, 40 μmol m^−2^ s^−1^). Explants were subcultured onto fresh medium of the same composition every month, and after one subculture cycle, healthy shoots were selected and transferred to the temporary immersion system (TIS) bioreactor.

### 4.2. Temporary Immersion System (TIS) Setup and Culture Conditions

*C. autumnale* and *C. bivonae* shoots were cultivated in TIS type RITA^®^ (Vitropic, Saint-Mathieu-de-Tréviers, France) filled with 200 mL liquid MS medium supplemented as described above. The pH was adjusted to 5.7 ± 0.1 before autoclaving at 121 °C for 20 min. Shoots were cultivated at 25 ± 2 °C under three immersion frequencies (15 min immersion followed by and 4 h, 8 h, and 12 h stand-by periods) under the photoperiod above-mentioned and they were subcultured every month onto fresh medium of the same composition (Figures S5a–c and S6a–c, respectively). Five explants per bioreactor were introduced, and three independent bioreactors per treatment were cultivated for each Colchicum species. Each bioreactor was considered an independent biological replicate. Metabolomic profiling was conducted using three biological replicates per treatment, corresponding to three independently operated bioreactors.

### 4.3. Biomass Growth Assessment

The growth of the shoots was monitored by calculating the growth index (GI), according to Dixon [56]:

(1) $$GI=\frac{FDB-IDB}{IDB},$$

where FDB is the final dry biomass, and IDB is the initial dry biomass.

The initial dry biomass (IDB) was estimated from a separate set of six shoots harvested at the time of inoculation, dried using a freeze dryer (Biobase Biodustry (Shandong) Co., Ltd., Jinan, Shandong, China), weighted, and averaged. The same average IDB value was applied to all immersion regimes for both species.

### 4.4. Metabolite Extraction and GC-MS Analysis

The extraction procedure for metabolite profiling and GC-MS analysis was carried out according to the methods described previously [57].

Untargeted metabolomics was conducted on samples from all three immersion frequencies (4 h, 8 h, and 12 h stand-by periods) for both *Colchicum* species. Metabolomic profiling was conducted using three biological replicates per treatment, corresponding to three independently operated bioreactors. For each biological replicate, three technical replicates were analysed by GC-MS. The coefficient of variation (CV) of the internal standards across all technical replicates remained below 15%, demonstrating satisfactory analytical reproducibility and instrument precision. In the present experimental design, analytical and biological variability were further minimised by pooling five explants within each bioreactor and by the high degree of environmental uniformity provided by the TIS culture conditions. Collectively, these measures enhanced the robustness and reliability of the obtained results. Under these conditions, three biological replicates were sufficient to detect the pronounced differences observed in this study.

### 4.5. Metabolite Identification and Quantification

Retention indices (RIs) were determined by injecting a homologous series of n-alkanes (C_10_–C_40;_ Sigma-Aldrich, St. Louis, MO, USA) under the same chromatographic conditions as the samples. RI values were calculated using the Automated Mass Spectral Deconvolution and Identification System (AMDIS) software (version 2.73) based on calibration with the standard n-alkane mixture. Metabolites were identified by comparing their mass spectra and calculated RIs with those of authentic standards (Sigma-Aldrich, St. Louis, MO, USA) and by matching spectral data with entries in the mass spectral library of the Mass Spectral Database, PC-Version 5.0, 2008 from the National Institute of Standards and Technology, Gaithersburg, MD, USA), as well as with reference data from the Golm Metabolome Database [58].

Quantification was performed using ribitol as an internal standard. Calibration curves were generated for representative compounds from each chemical class using authentic standards. Metabolites lacking corresponding standards were quantified on a relative basis using the response factor of the closest structural analogue. Metabolite concentrations were expressed as milligrams per gram of dry matter (mg/g DM). Detailed information on identified compounds, including retention times, characteristic ions, and levels of identification confidence, is provided in Supplementary Table S1.

### 4.6. Data Preprocessing and Statistical Analysis

Raw GC-MS data were processed using ChemStation software F01 032357 (Agilent Technologies, Santa Clara, CA, USA) for peak detection, integration, and alignment. Metabolites present in fewer than 50% of samples within any group were excluded from downstream analysis. Missing values were imputed using half of the minimum positive value detected for each metabolite. To address the wide dynamic range of metabolite concentrations and ensure equitable contribution to multivariate models, the data were log_10_-transformed and subjected to unit variance (UV) scaling (mean-centred and divided by the standard deviation of each variable) [59]. Principal component analysis (PCA) was performed as an unsupervised exploratory method to visualise overall sample clustering and detect outliers. Partial least squares discriminant analysis (PLS-DA) was employed as a supervised method to maximise the separation between species and identify discriminatory metabolites. The quality of the PLS-DA model was assessed by cross-validation using 100 permutation tests [60]. Variable importance in projection (VIP) scores were calculated to rank metabolites by their contribution to the discrimination; metabolites with VIP > 1.0 were considered significant biomarkers [61]. Hierarchical clustering analysis (HCA) was performed using Ward’s linkage method with Euclidean distance as the similarity measure. The resulting heatmap was generated using the heatmap package in MetaboAnalyst (version 6.0) [62]. Univariate comparisons between species were performed using the Student’s *t*-test with Benjamini–Hochberg correction for multiple testing (false discovery rate < 0.05).

## 5. Conclusions

This study demonstrates that temporary immersion systems, particularly the optimised regime of 15 min immersion followed by a 4 h stand-by period, provide an effective platform for the cultivation of *C. autumnale* and *C. bivonae*, supporting robust biomass proliferation and stable colchicine biosynthesis. GC-MS-based metabolomics enabled the identification and quantification of 46 distinct metabolites and revealed differences in metabolite profiles between the two *Colchicum* species, particularly in compounds related to carbon and nitrogen metabolism. *C. bivonae* exhibited a pattern characterised by higher carbohydrate abundance, high glycolytic flux (elevated G6P), and efficient nitrogen storage (asparagine), which correlated with superior colchicine yields (0.25 mg/g DB) and metabolic consistency. In contrast, *C. autumnale* exhibited a metabolic profile characterised by enhanced TCA cycle activity (elevated citric acid) and the accumulation of stress-associated metabolites (GABA, pyroglutamic acid), resulting in lower colchicine production (0.19 mg/g DB) and greater metabolic variability.

Integration of univariate (volcano plot, SAM) and multivariate (PLS-DA and hierarchical clustering) statistical approaches identified glucose-6-phosphate, citric acid, asparagine, and GABA as the primary metabolic biomarkers differentiating the two species. These findings establish a basis for the rational optimisation of TIS-based production systems for colchicine and position *C. bivonae* as a promising candidate for biotechnological exploitation. The metabolomic framework developed here can be extended to other medicinal plants and in vitro culture systems to guide process optimisation and enhance the sustainable production of high-value secondary metabolites.

## Figures and Tables

**Table 1:** Analyses of variance showing the importance of the effects of species and immersion frequency on the growth index and colchicine production in TISs.

Response	F-Value	*p*-Value
Growth index		
*Immersion*	159.62	0
*Species*	73.28	0
Immersion * Specie	13.12	0.003
Colchicine		
*Immersion*	0.33	0.572
*Species*	130.37	0
Immersion * Specie	8.93	0.01

The asterisk (*) indicates an interaction effect between the two factors.

**Table 2:** Key discriminatory metabolites with variable importance in projection (VIP) scores in *C. bivonae* and *C. autumnale* cultivated in a temporary immersion system with an optimised immersion frequency of 15 min immersion and 4 h stand-by periods. Metabolites with VIP > 1.0 were considered significant biomarkers for species differentiation. Fold change is calculated as *C. bivonae*/*C. autumnale.* DM = dry matter.

Metabolite	VIP Score	Fold Change (Cb/Ca)	Metabolic Class
Glucose-6-phosphate	2.01	5.11	Sugar phosphate
Citric acid	1.85	0.39	Organic acid (TCA)
Asparagine	1.67	2.05	Amino acid (N storage)
GABA	1.52	0.25	Stress metabolite
Sucrose	1.48	1.43	Disaccharide
Pyroglutamic acid	1.41	0.6	Stress-related amino acid
Malic acid	1.35	0.59	Organic acid (TCA)
Glycine	1.28	1.49	Amino acid
Colchicine	1.22	1.32	Alkaloid
Salicylic acid	1.18	2.33	Phenolic compound

**Table 3:** Summary of key metabolic features distinguishing *C. bivonae* and *C. autumnale*.

Metabolic Feature	*C. bivonae*	*C. autumnale*
Carbon Strategy	Carbohydrate-rich (sucrose, glucose, sorbitol)	Organic acid-rich (citrate, malate, succinate)
Nitrogen Strategy	Asparagine, glycine, glutamic acid	Pyroglutamic acid, GABA, serine
Energy Metabolism	High glycolytic flux (G6P elevation)	High TCA cycle activity
Secondary Metabolism	Higher colchicine, enriched phenolic acids	Lower colchicine, fewer phenolic acids
Metabolic Consistency	Tight clustering, stable metabolome	Broader dispersion, variable sugar pools
Stress Signature	Low	High (GABA, pyroglutamic acid)

## Data Availability

The data presented in this study are available within the article and its Supplementary Materials.

## Supplementary Materials

The following supporting information can be downloaded at: https://www.mdpi.com/article/10.3390/plants15111710/s1, Figure S1a,b: Response optimisation plots of stand-by and immersion times for theoretically maximal production of colchicine and growth index by *Colchicum autumnale* and *Colchicum bivonae* cultivated in TISs; Figure S2a,d: Pareto charts; Figure S2b,c,e,f: Factorial plots; Figure S3: *C. bivonae* and *C. autumnale* corms; Figure S4: Initiation of in vitro cultures, a—main bud; b—lateral bud; c—excision of explants; d—inoculated explant placed on nutrient medium in culture tubes; Figure S5a–c: *C. autumnale* in TIS at 4, 8, and 12 h stand-by/15 min immersion periods; Figure S6a–c: *C. bivonae* in TISs at 4, 8 and 12 h stand-by/15 min immersion periods; Table S1: Number of introduced explants; natural habitats of collected samples of *C. autumnale* and *C. bivonae;* GC-MS data of identified metabolites and statistical data.

## Abbreviations

The following abbreviations are used in this manuscript:

BAP	6-Benzylaminopurine
DB	Dry biomass
FDR	False discovery rate
FDB	Final dry biomass
FW	Fresh weight
G6P	Glucose-6-phosphate
GABA	γ-Aminobutyric acid
GC-MS	Gas chromatography-mass spectrometry
GI	Growth index
HCA	Hierarchical clustering analysis
IDB	Initial dry biomass
MS	Murashige and Skoog
NAA	α-Naphthaleneacetic acid
PCA	Principal component analysis
PLS-DA	Partial least squares discriminant analysis
RI	Retention index
RITA^®^	Recipient for Temporary Immersion (bioreactor)
SAM	Significance analysis of metabolomics
TCA	Tricarboxylic acid
TIS	Temporary immersion system
UV	Unit variance
VIP	Variable importance in projection
