# Supplementary material for: Immersion Frequency Optimisation and Species-Specific Metabolic Profiles of Colchicum autumnale and Colchicum bivonae in Temporary Immersion Systems
Source: Plants (Basel). 2026 May 31;15(11):1710. doi: 10.3390/plants15111710 (PMC13259014; doi:10.3390/plants15111710)
Supplement: Supplementary file 1 [file plants-15-01710-s001.zip › Supplementary Figures S1_S6.pdf]

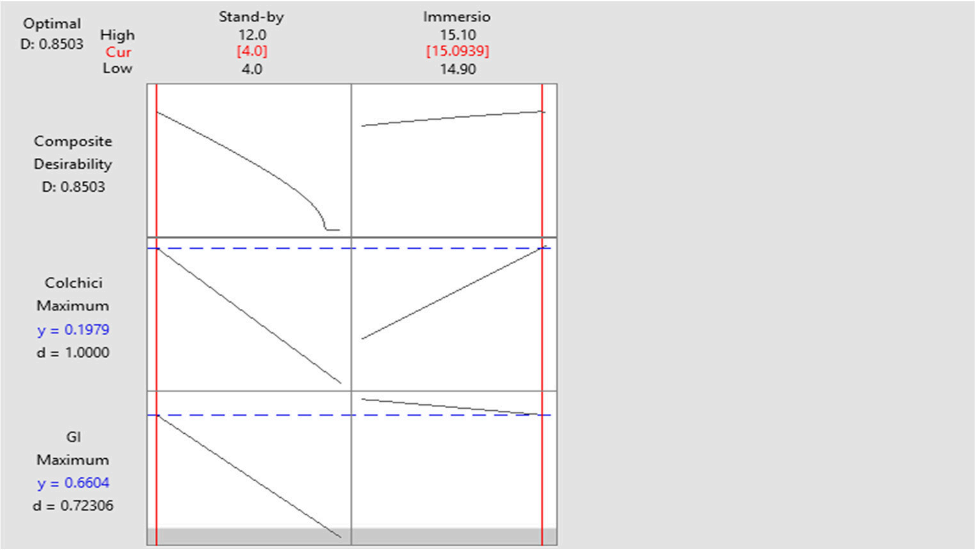

Figure S1a. Response optimization plots of stand-by and immersion times for theoretically maximal production of colchicine and Growth index by *C. autumnale* cultivated in TIS.

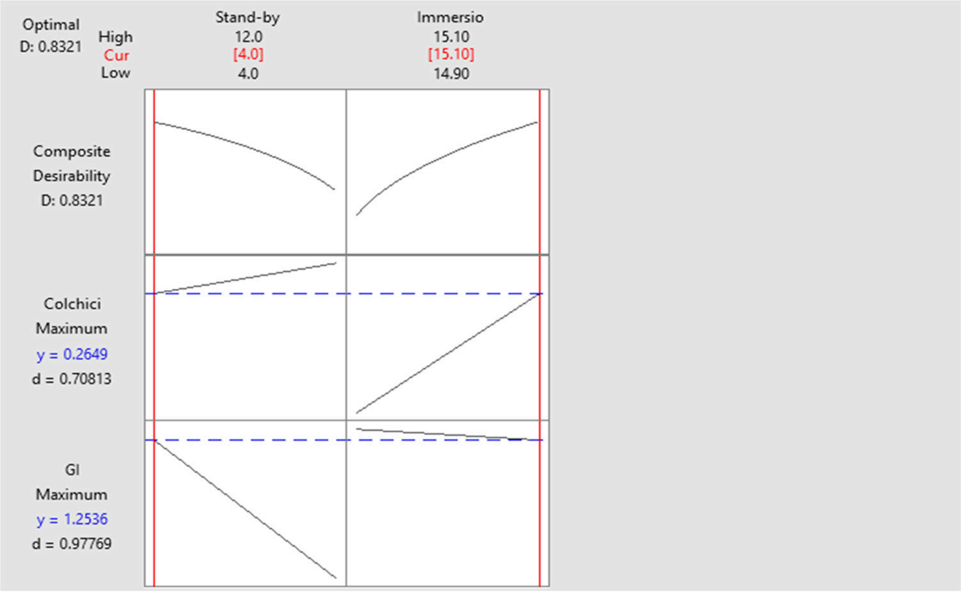

Figure S1b. Response optimization plots of stand-by and immersion times for theoretically maximal production of colchicine and Growth index by *C. bivonae* cultivated in TIS.

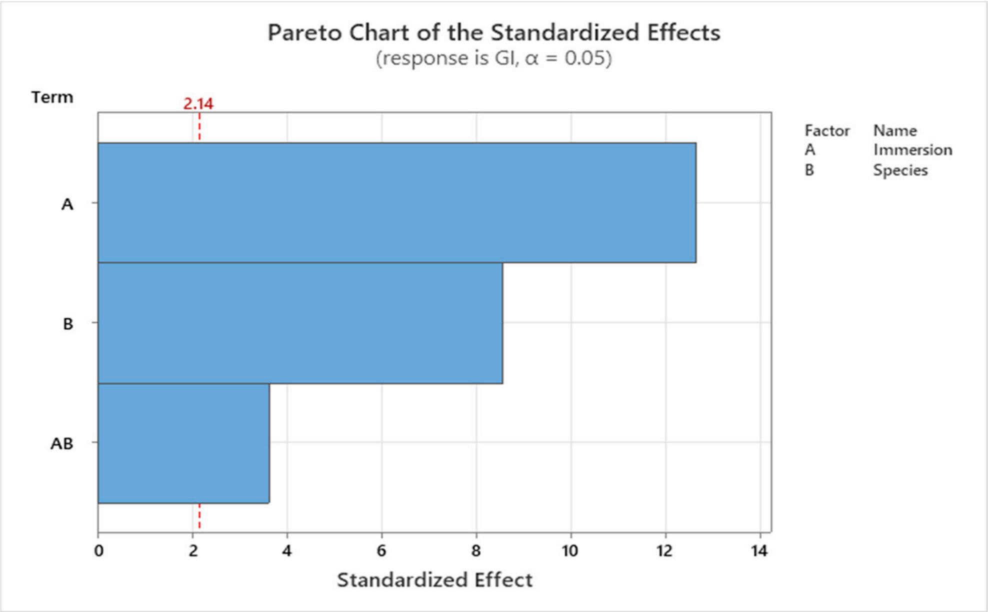

Figure S2a. Pareto chart showing the magnitude and the importance of the effects (immersion regimes and species) on the Growth Index at the significance level of 0.05.

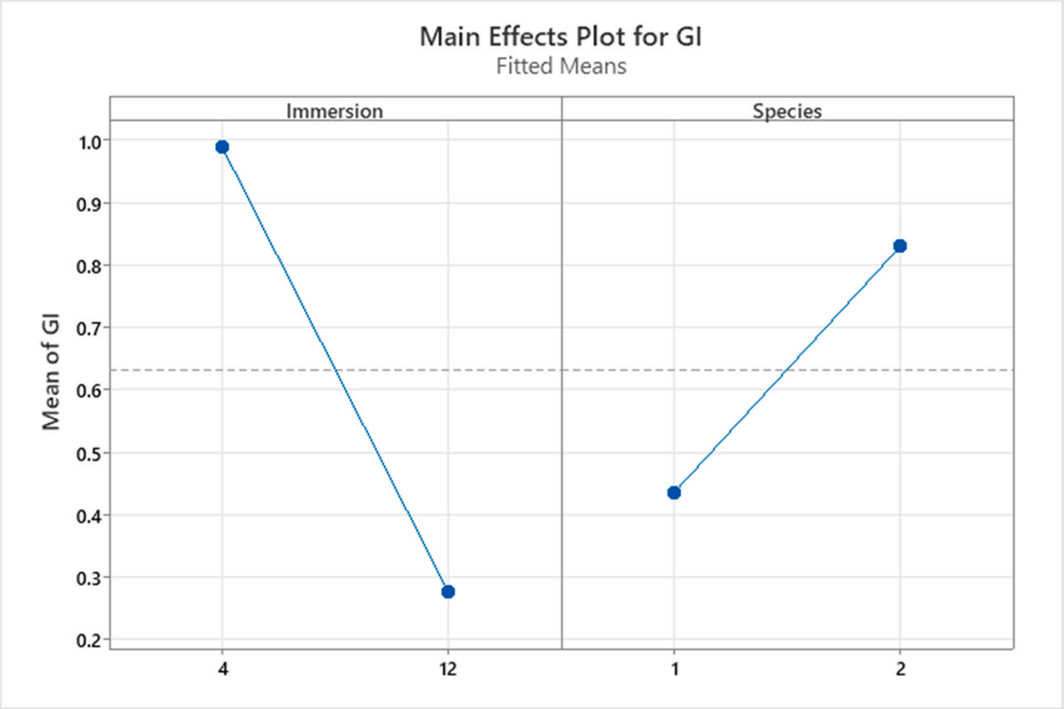

Figure S2b. Factorial plots visualizing the relationship between factors (immersion regimes and species) and a response variable (Growth Index). The lines showing the main effects and interactions of single factors.

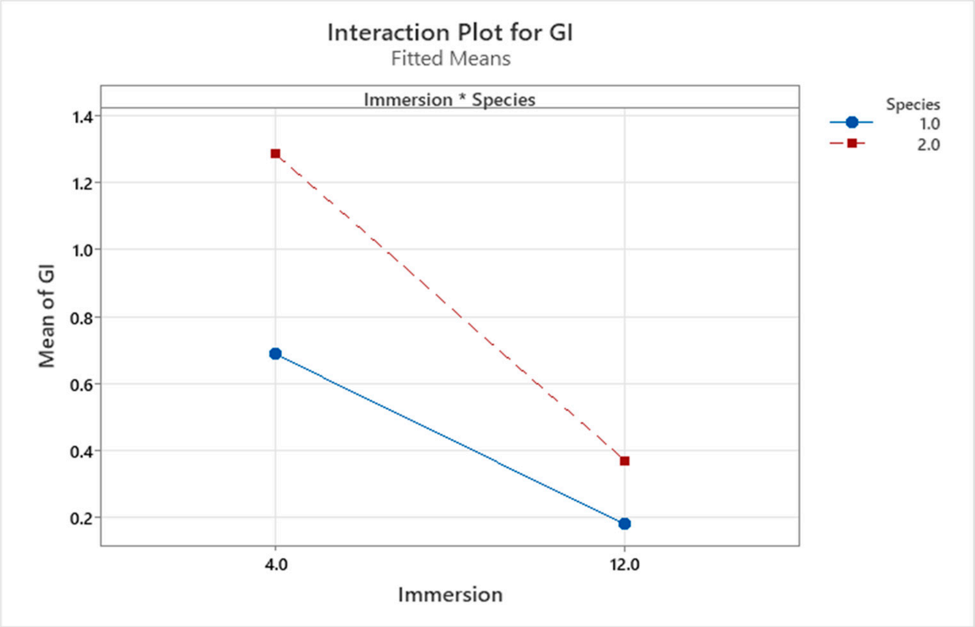

Figure S2c. Factorial plots visualizing the relationship of two-way interactions of factors (immersion regimes and species) and a response variable (Growth Index).

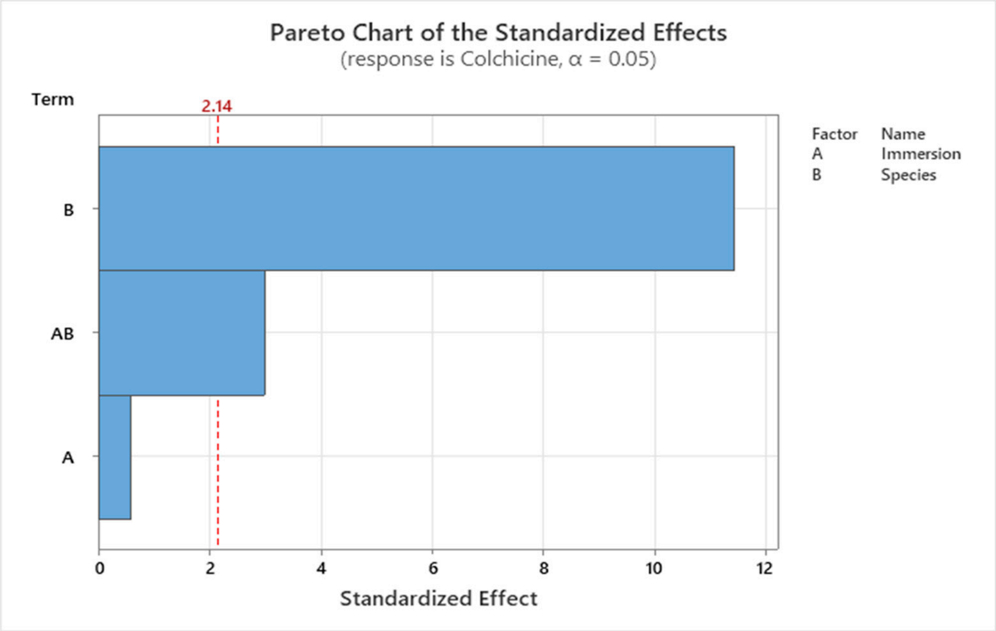

Figure S2d. Pareto chart showing the magnitude and the importance of the effects (immersion regimes and species) on the colchicine (mg/g dw) at the significance level of 0.05.

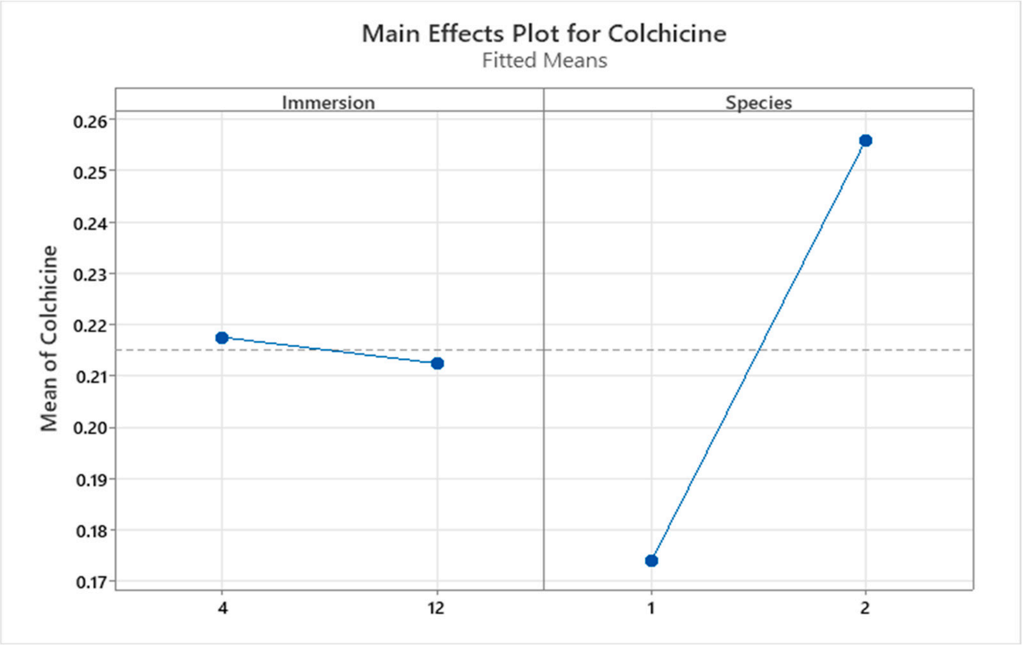

Figure S2e. Factorial plots visualizing the relationship between factors (immersion regimes and species) and a response variable (colchicine, mg/g dw). The lines showing the main effects and interactions of single factors.

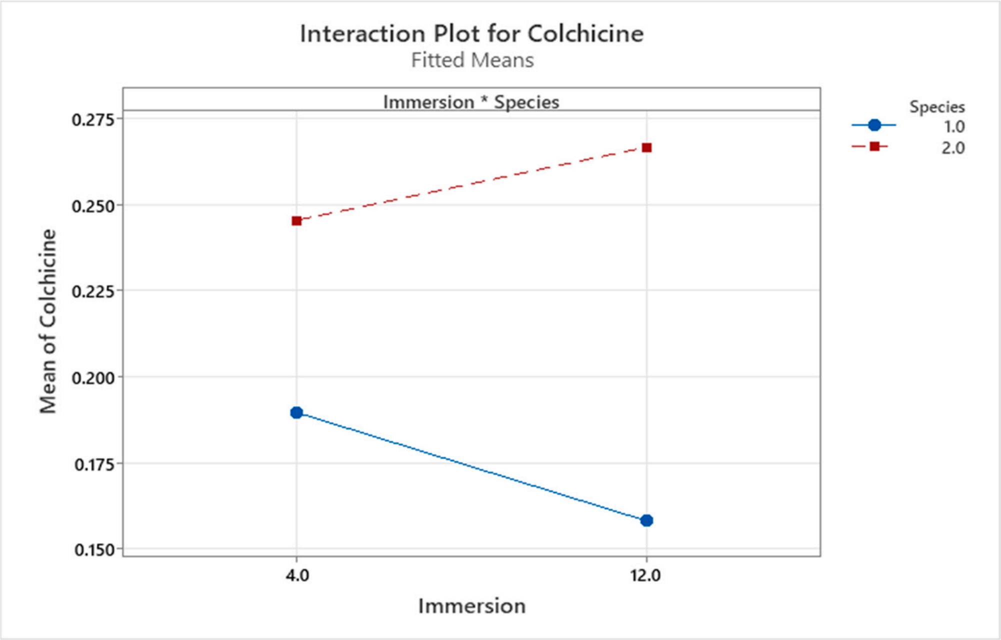

Figure S2f. Factorial plots visualizing the relationship of two-way interactions of factors (immersion regimes and species) and a response variable (colchicine, mg/g dw).

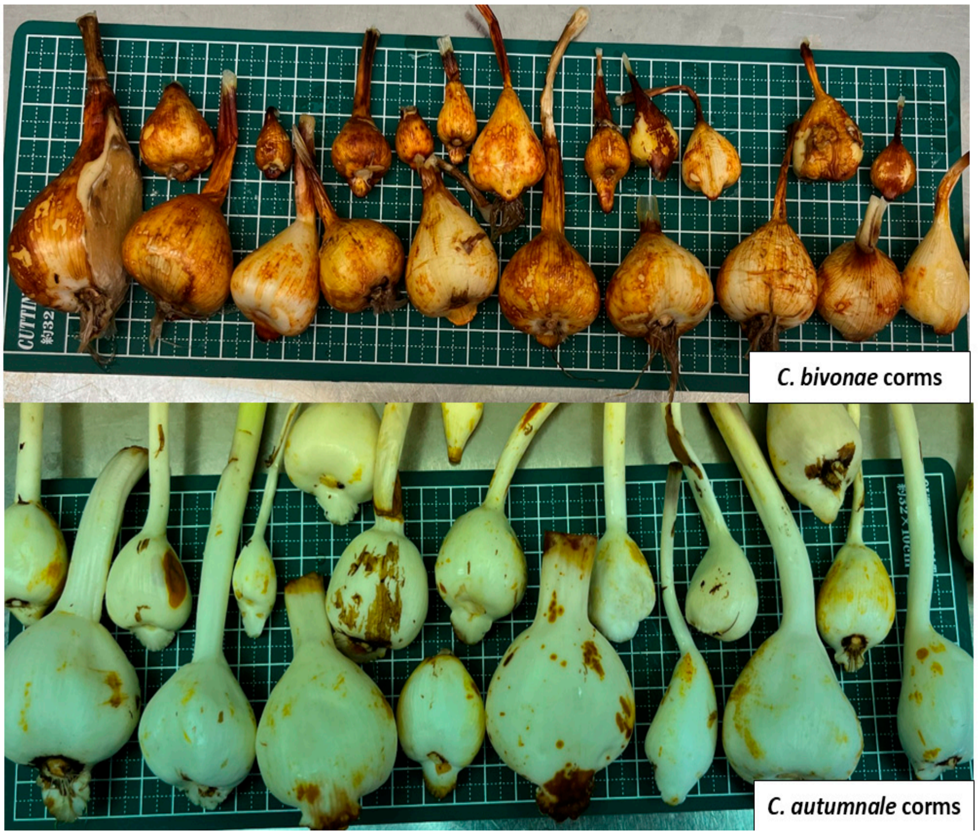

Figure S3: *C. bivonae* and *C. autumnale* corms.

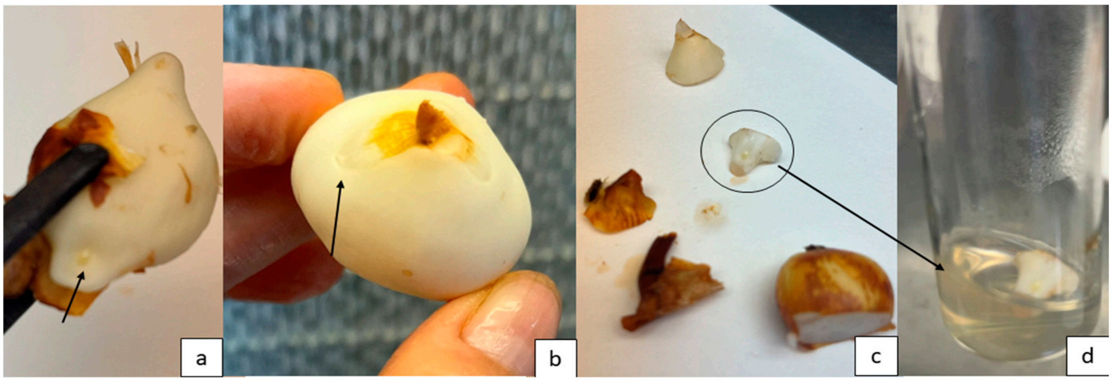

Figure S4: Initiation of *in vitro* cultures of *Colchicum* species: a - main bud; b - lateral bud; c - excision of explants; d - inoculated explant placed on nutrient medium in culture tubes

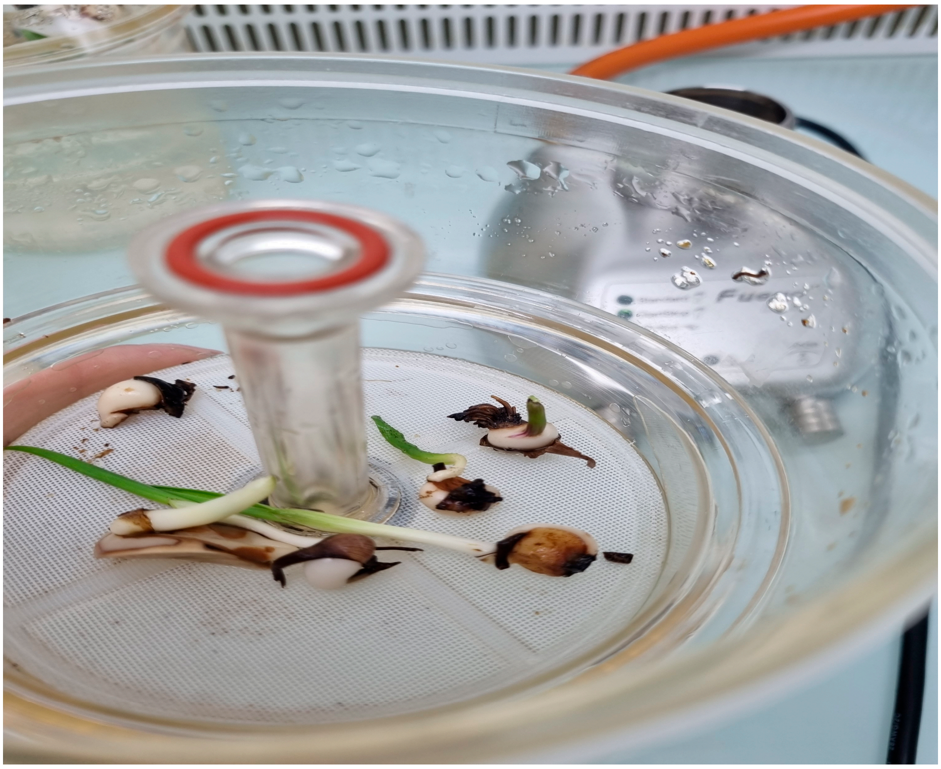

Figure S5a: *C. autumnale* in temporary immersion system (TIS) at 4 h stand-by/15 min immersion periods.

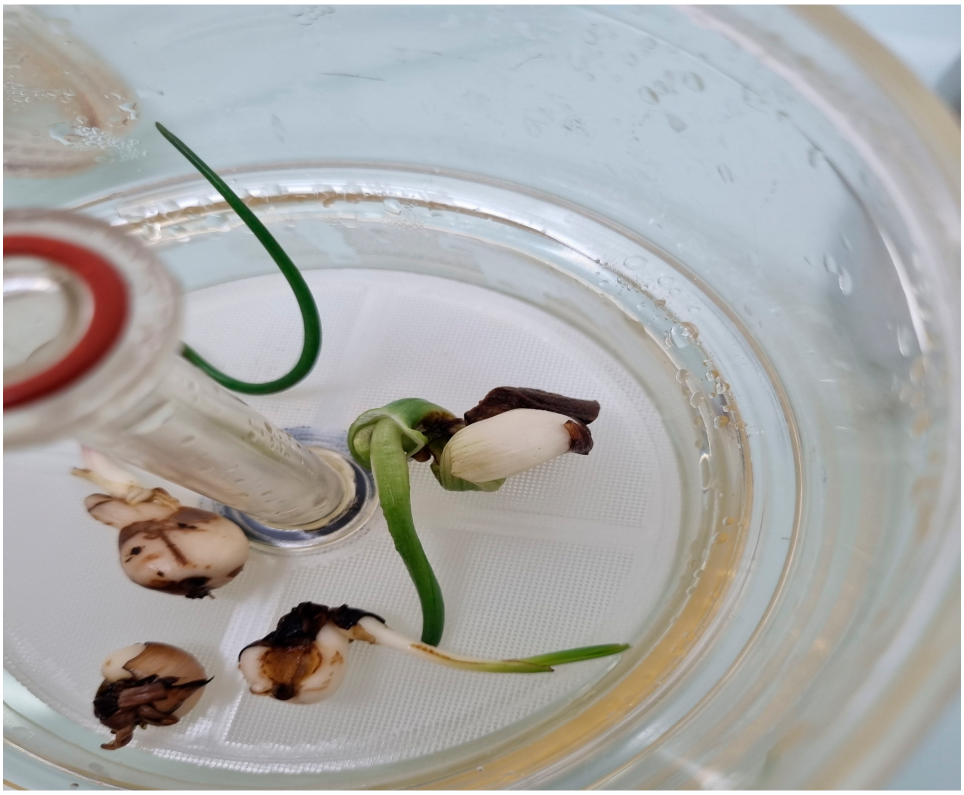

Figure S5b: *C. autumnale* in temporary immersion system (TIS) at 8 h stand-by/15 min immersion periods.

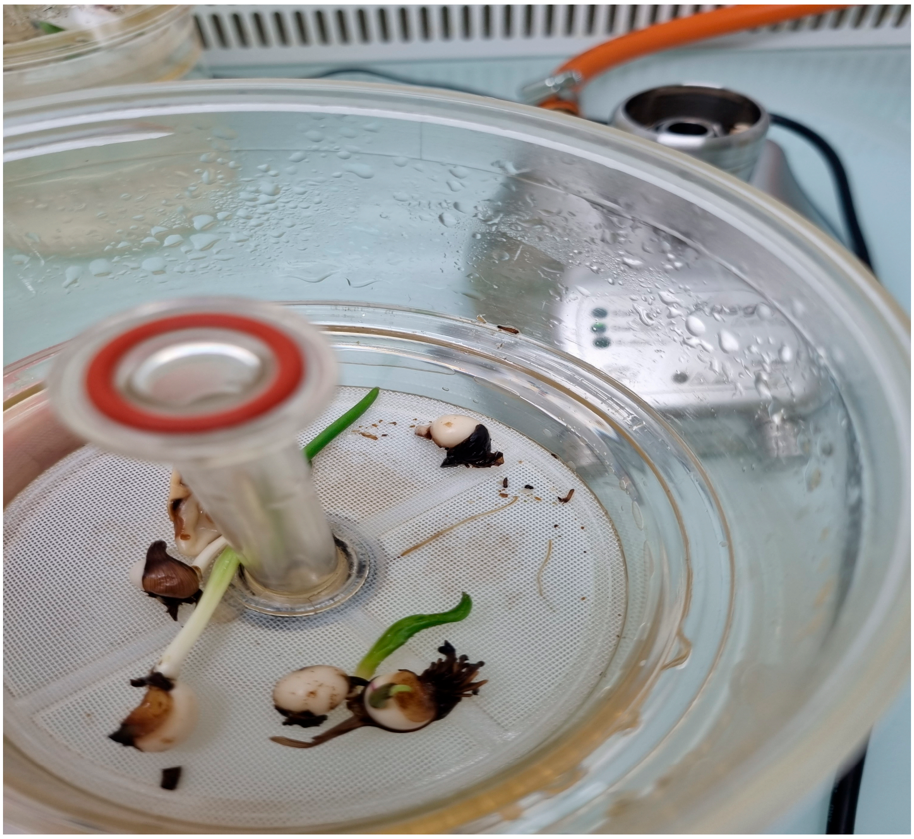

Figure S5c: *C. autumnale* in temporary immersion system (TIS) at 12 h stand-by/15 min immersion periods.

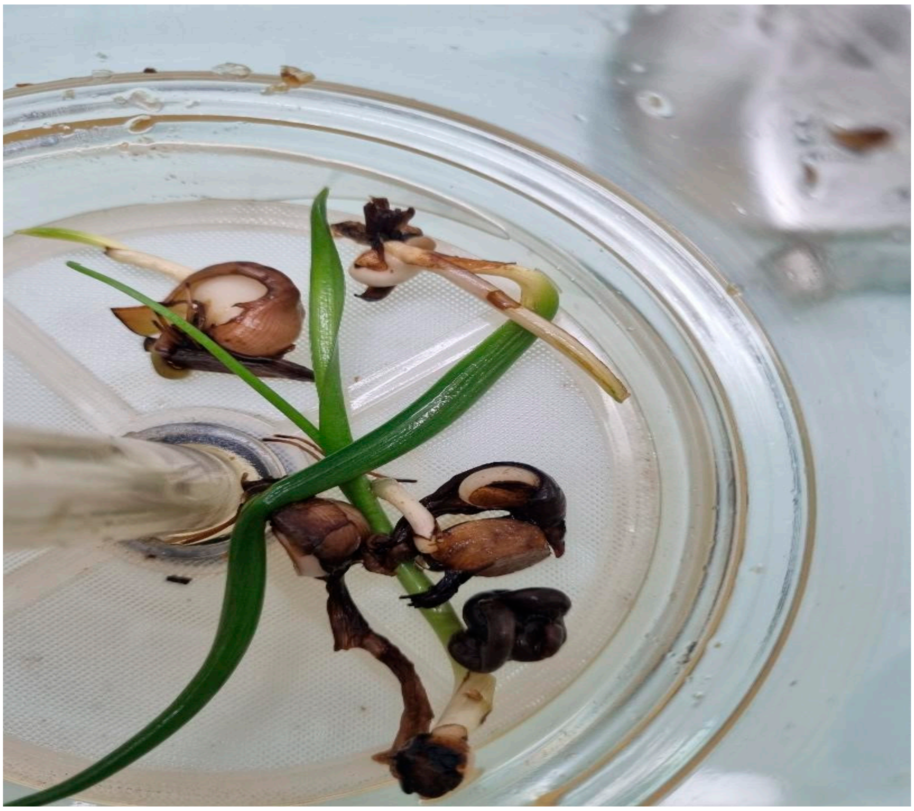

Figure S6a: *C. bivonae* in temporary immersion system (TIS) at 4 h stand-by/15 min immersion periods.

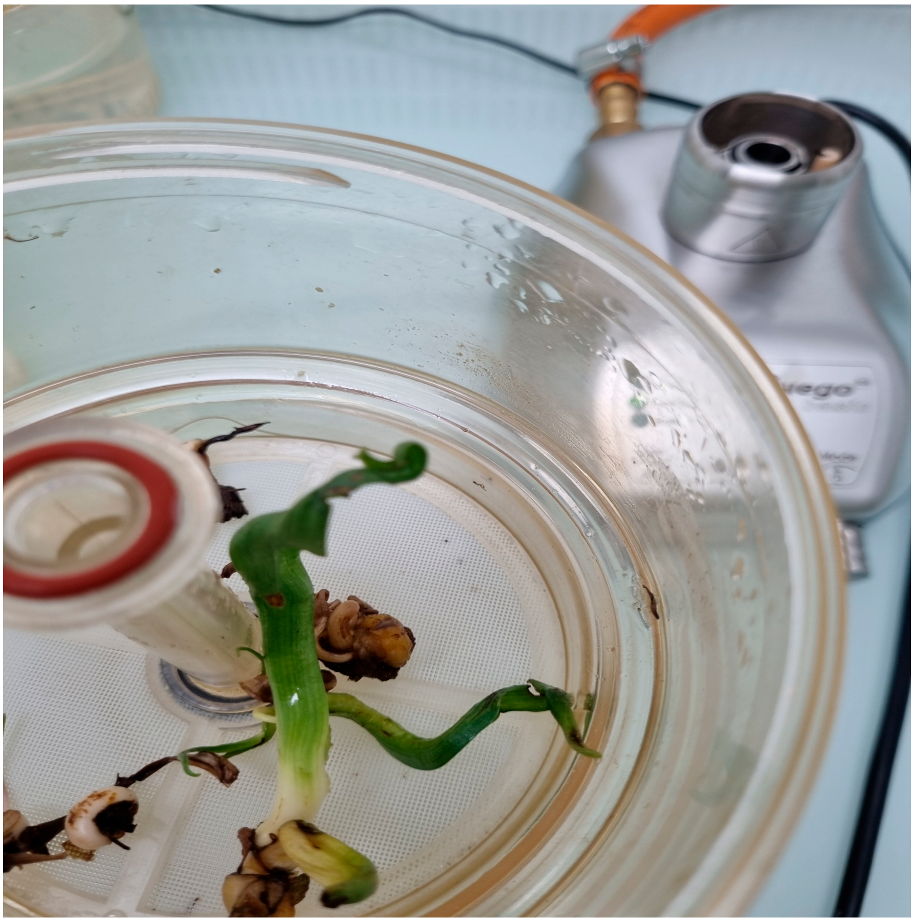

Figure S6b: *C. bivonae* in temporary immersion system (TIS) at 8 h stand-by/15 min immersion periods.

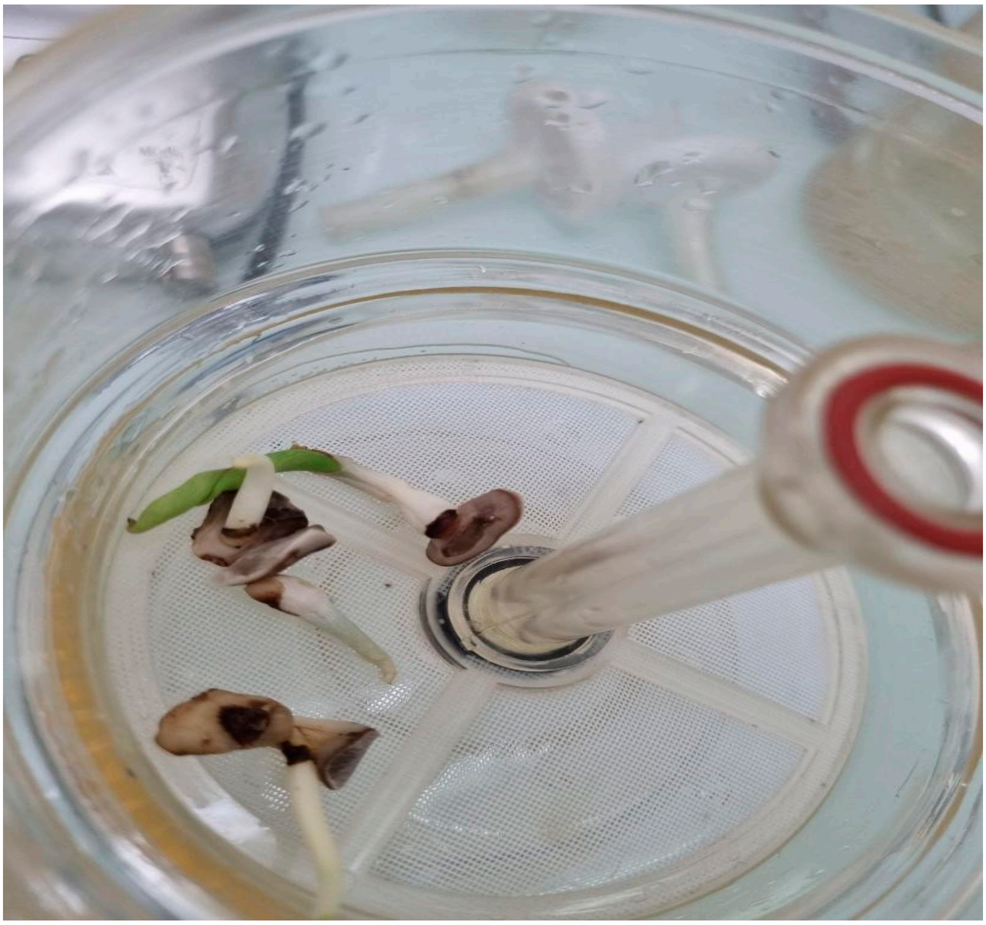

Figure S6c: *C. bivonae* in temporary immersion system (TIS) at 12 h stand-by/15 min immersion periods.
