# Supplementary figures and images for: Immersion Frequency Optimisation and Species-Specific Metabolic Profiles of Colchicum autumnale and Colchicum bivonae in Temporary Immersion Systems
Source: Plants (Basel). 2026 May 31;15(11):1710. doi: 10.3390/plants15111710 (PMC13259014; doi:10.3390/plants15111710)

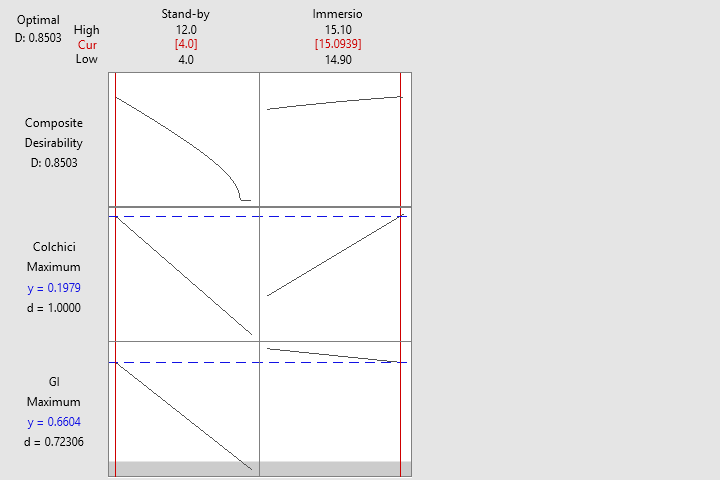

Supplement: Supplementary file 1 [file plants-15-01710-s001.zip › Fig. S1a.png]

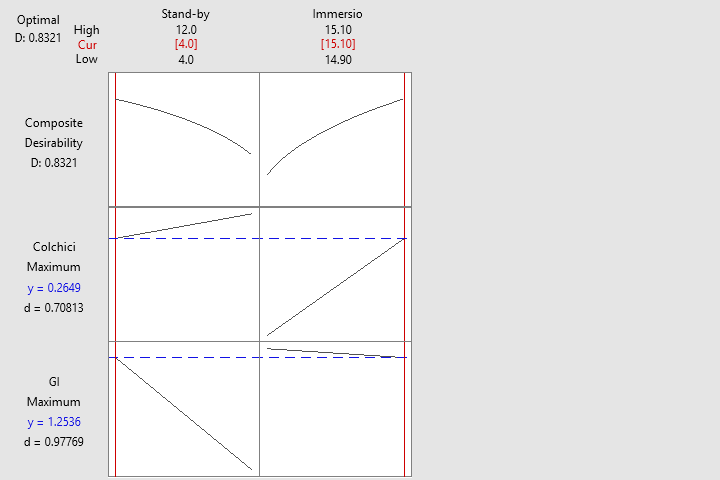

Supplement: Supplementary file 1 [file plants-15-01710-s001.zip › Fig. S1b.png]

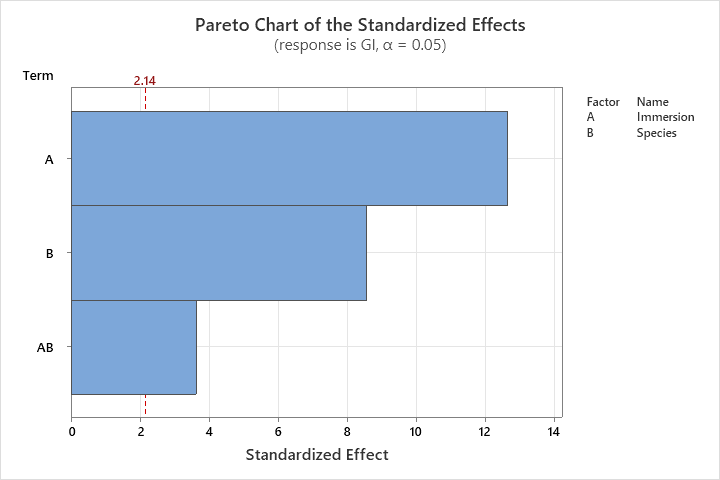

Supplement: Supplementary file 1 [file plants-15-01710-s001.zip › Fig. S2a.png]

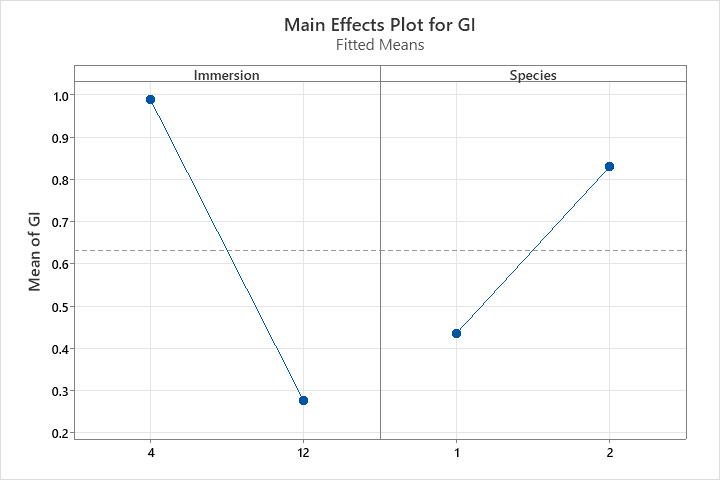

Supplement: Supplementary file 1 [file plants-15-01710-s001.zip › Fig. S2b.png]

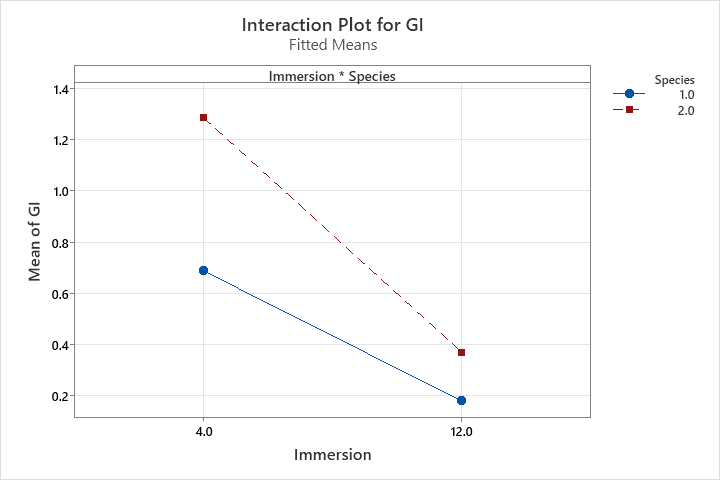

Supplement: Supplementary file 1 [file plants-15-01710-s001.zip › Fig. S2c.png]

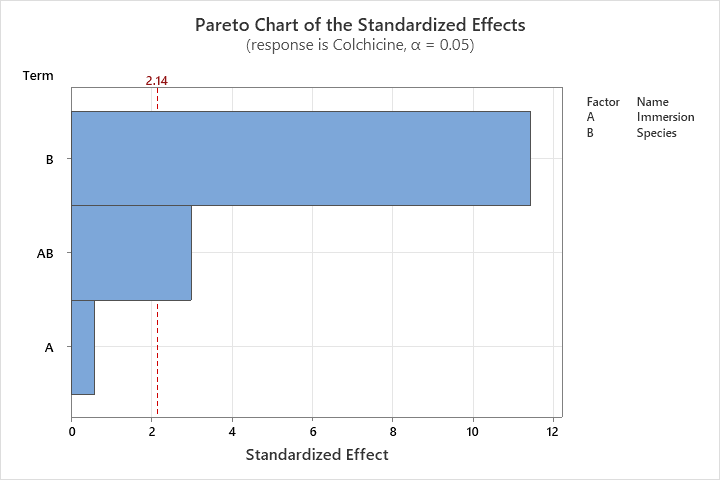

Supplement: Supplementary file 1 [file plants-15-01710-s001.zip › Fig. S2d.png]

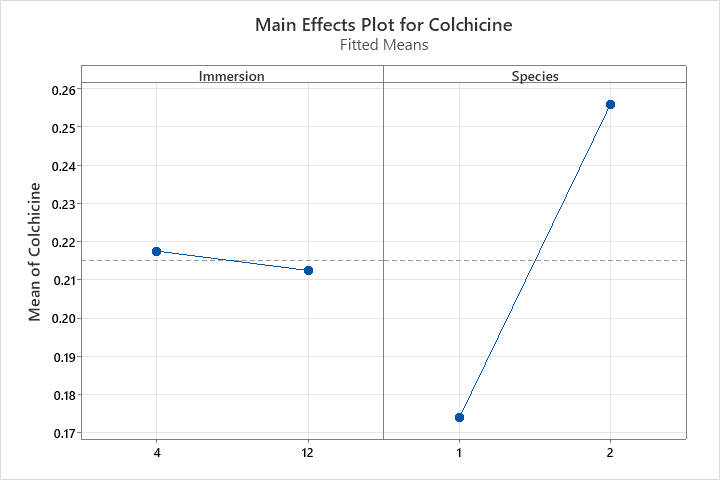

Supplement: Supplementary file 1 [file plants-15-01710-s001.zip › Fig. S2e.png]

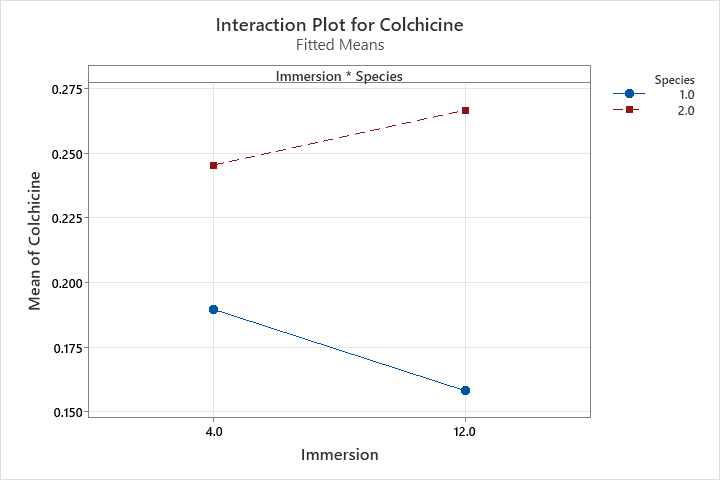

Supplement: Supplementary file 1 [file plants-15-01710-s001.zip › Fig. S2f.png]

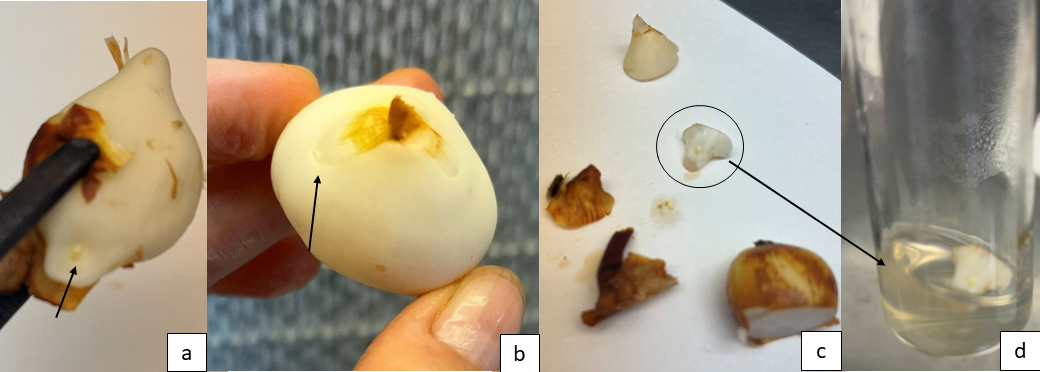

Supplement: Supplementary file 1 [file plants-15-01710-s001.zip › Figure S3_Ca and Cb corms.PNG]

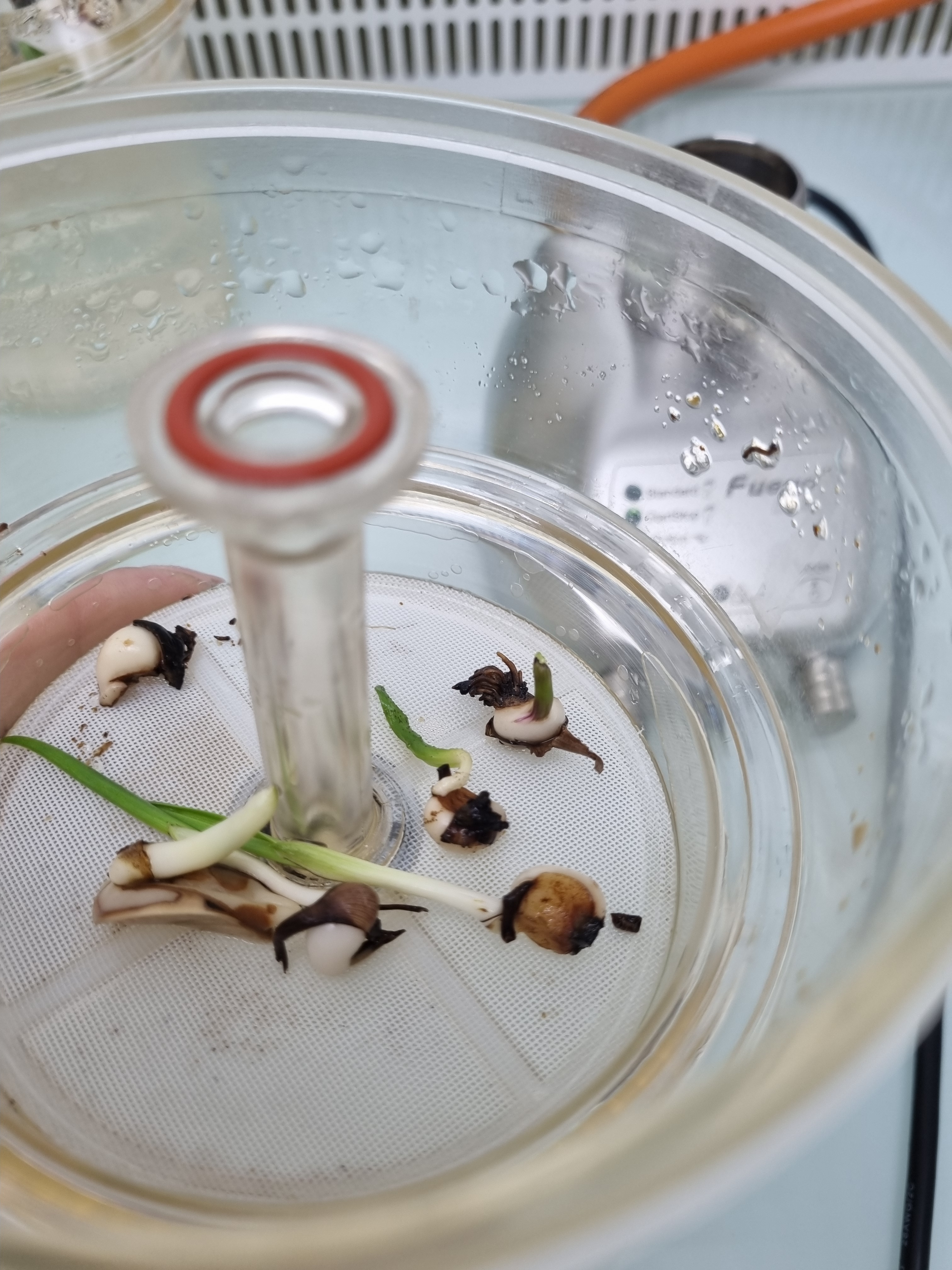

Supplement: Supplementary file 1 [file plants-15-01710-s001.zip › Figure S4_Initiation of in vitro cultures.png]

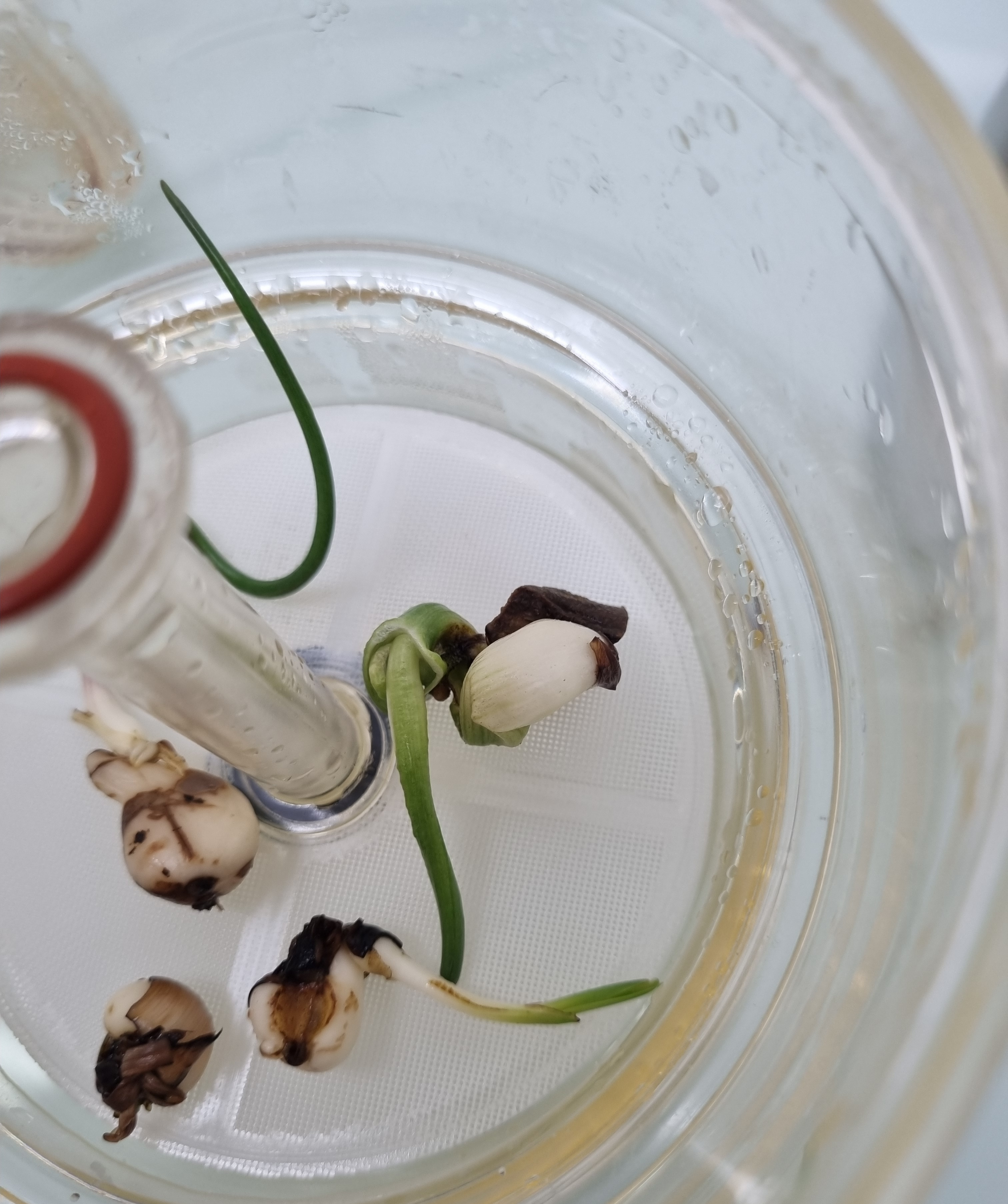

Supplement: Supplementary file 1 [file plants-15-01710-s001.zip › Figure S5a_C. autumnale_4_15.png]

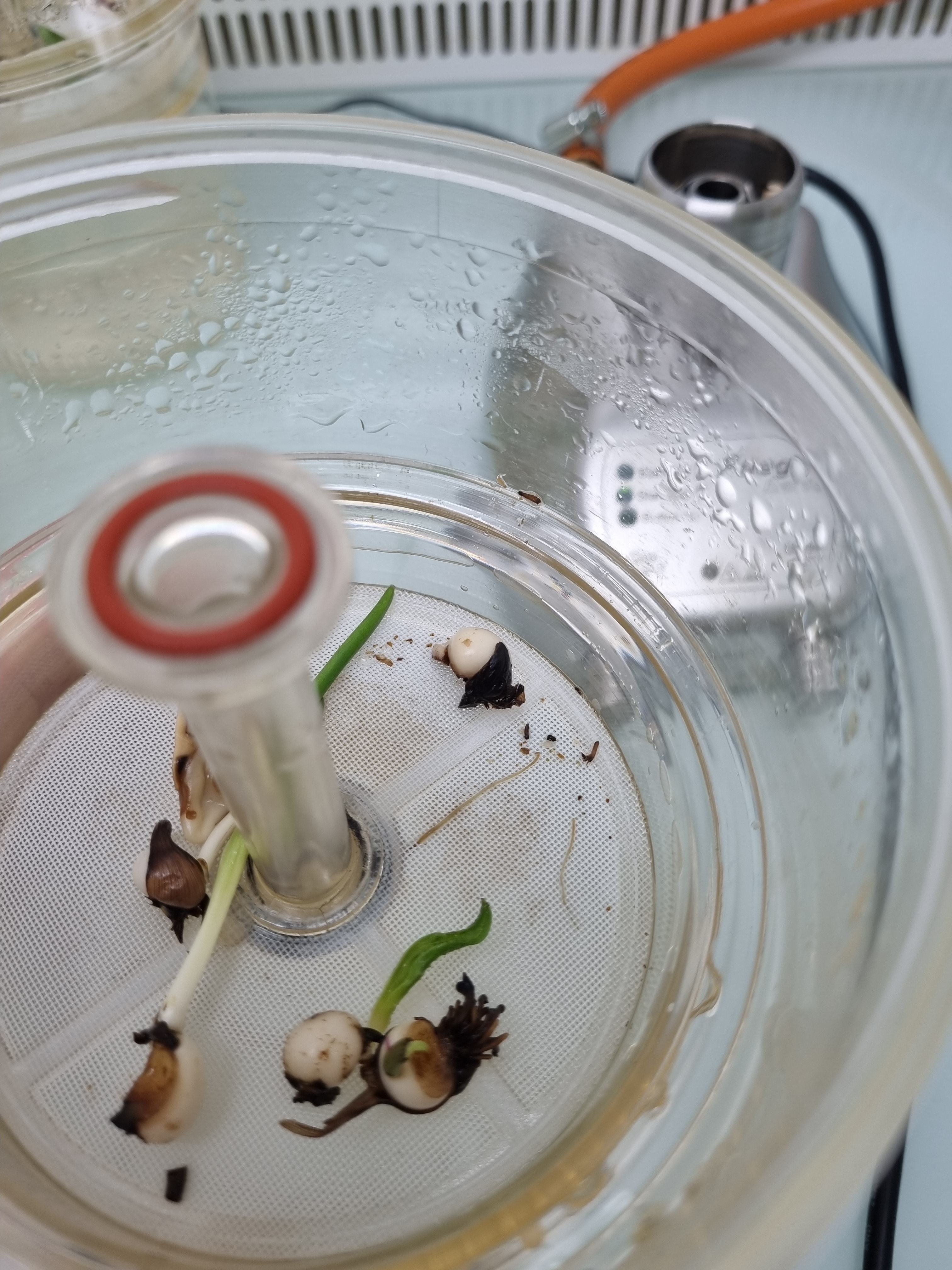

Supplement: Supplementary file 1 [file plants-15-01710-s001.zip › Figure S5b_C. autumnale_8_15.png]

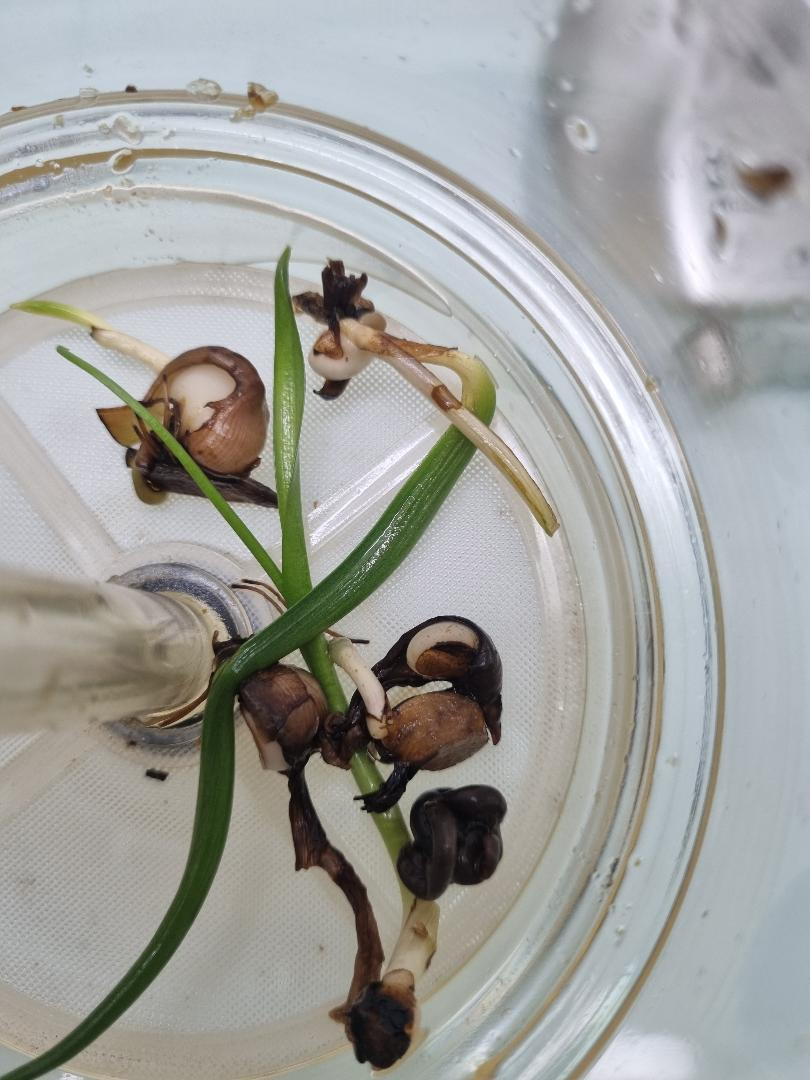

Supplement: Supplementary file 1 [file plants-15-01710-s001.zip › Figure S5c_C. autumnale_12_15.png]

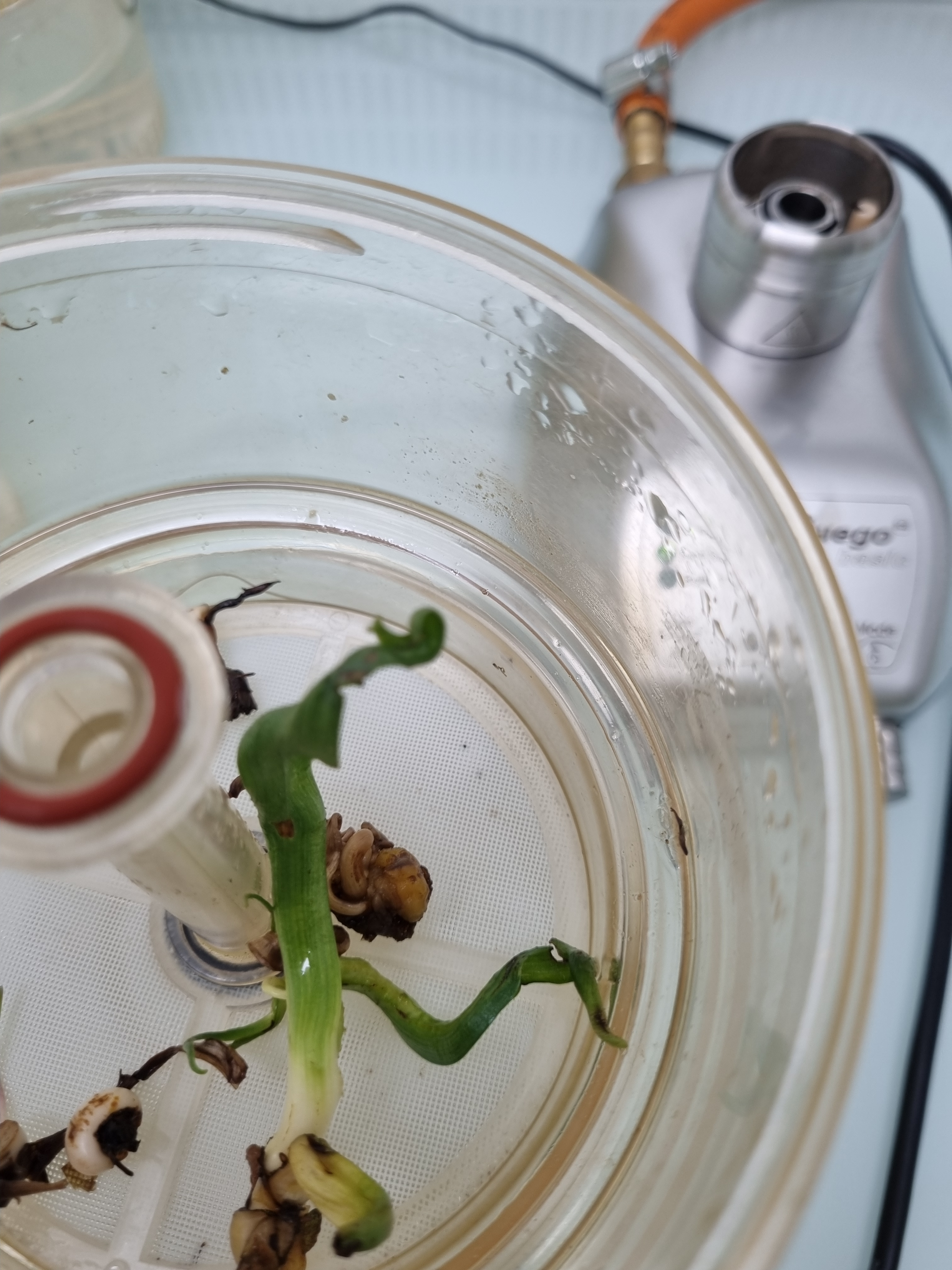

Supplement: Supplementary file 1 [file plants-15-01710-s001.zip › Figure S6a_C.bivonae_4_15.png]

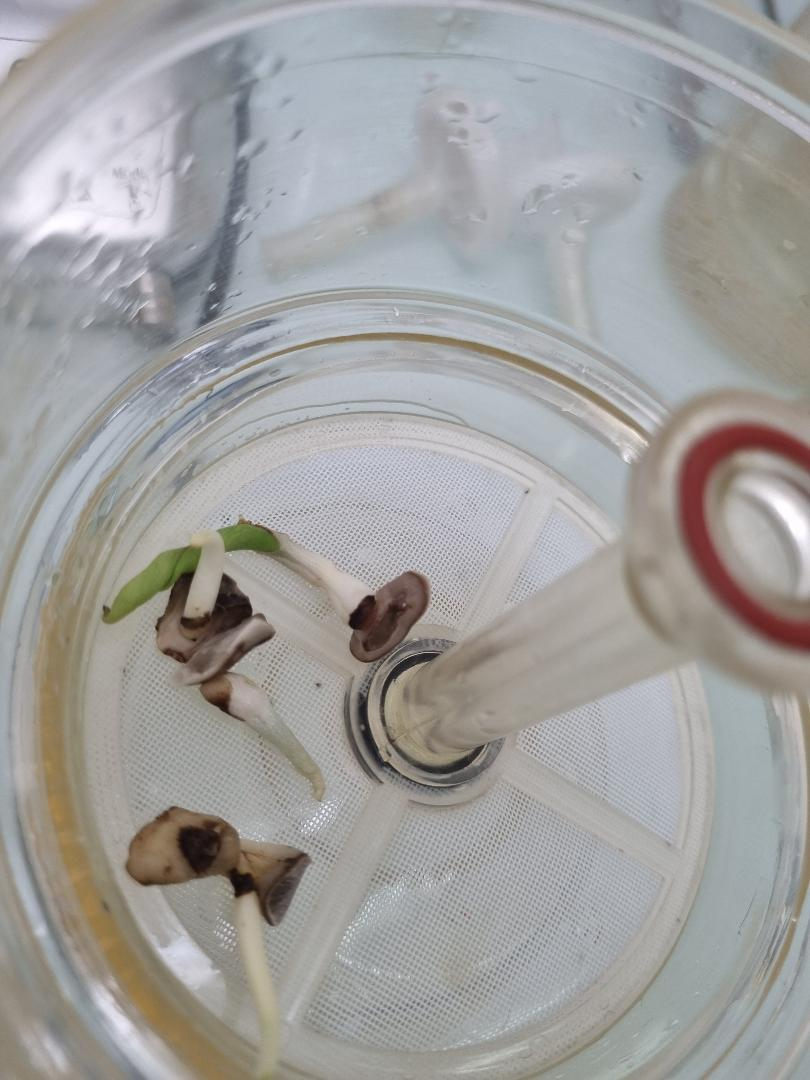

Supplement: Supplementary file 1 [file plants-15-01710-s001.zip › Figure S6b_C. bivonale_8_15.png]
